# Designing Versatile Polymers for Lithium-Ion Battery Applications: A Review

**DOI:** 10.3390/polym14030403

**Published:** 2022-01-20

**Authors:** Beatriz Arouca Maia, Natália Magalhães, Eunice Cunha, Maria Helena Braga, Raquel M. Santos, Nuno Correia

**Affiliations:** 1 Materials and Composite Structures Unit, Institute of Science and Innovation in Mechanical and Industrial Engineering (INEGI), 4000-014 Porto, Portugal; bmaia@inegi.up.pt (B.A.M.); nmagalhaes@inegi.up.pt (N.M.); rmsantos@inegi.up.pt (R.M.S.); ncorreia@inegi.up.pt (N.C.); 2 LAETA—Associated Laboratory of Energy, Transports and Aeronautics, 4200-265 Porto, Portugal; mbraga@fe.up.pt; 3 Chemical Engineering Department, FEUP—Faculty of Engineering, University of Porto, 4200-265 Porto, Portugal; 4 Engineering Physics Department, FEUP—Faculty of Engineering, University of Porto, 4200-265 Porto, Portugal

**Keywords:** polymer electrolytes, lithium-ion battery, energy storage, composites

## Abstract

Solid-state electrolytes are a promising family of materials for the next generation of high-energy rechargeable lithium batteries. Polymer electrolytes (PEs) have been widely investigated due to their main advantages, which include easy processability, high safety, good mechanical flexibility, and low weight. This review presents recent scientific advances in the design of versatile polymer-based electrolytes and composite electrolytes, underlining the current limitations and remaining challenges while highlighting their technical accomplishments. The recent advances in PEs as a promising application in structural batteries are also emphasized.

## 1. Introduction

Global development is highly dependent on energy. Considering the impact of fossil fuels, global warming, and widespread pollution, the need for green, renewable, and alternative energy sources and storage systems is vital [1]. The cell of an electric battery is seemingly simple and easy to implement. It is based on the combination of two electrodes separated by an electrolyte to generate and store electric energy through a mechanism involving electrochemical reactions, with a spontaneous discharge and a charge requiring external electrical work. The first is driven by the necessity to align the electrochemical potentials of the electrodes and the latter serves the creation of the bias. Several approaches have been developed to optimize cells for different final applications and performance requirements [2].

At the end of the 20th century, and driven by the emerging market for wireless technologies, the demand for efficient and safe rechargeable batteries increased and lithium-based batteries became the go-to solution to fulfil most requirements [3]. Lithium (Li), an alkali metal, offers the best energy capacity and long-term life due to its high theoretical capacity. However, due to market pressures, it has become increasingly scarce and features in most critical raw materials lists. Continuous progress in Li-based batteries has been promoted by the intensive research carried out by scientists and engineers and a globally expanding need for better systems, where innovative battery components contribute significantly to producing and storing electrical energy. Nonetheless, it is necessary to highlight the crucial importance of the electrolyte and its influence on the capacity, stability, and operating conditions of Li-ion batteries (LIBs) [4,5,6]. Electrolyte performance has a direct impact on the working temperature, safety, cyclability, and cell capacity of the battery [7].

Depending on their physical state, electrolytes can be classified as liquid or solid. Solid electrolytes are typically classified in two subtypes: (i) organic (polymer-based) and (ii) inorganic. On the other hand, polymer electrolytes can be subdivided into solid-polymer electrolytes (SPEs) and gel-polymer electrolytes (GPEs). In the former, the polymeric matrix is a solid ionic conductor itself, and the latter comprises the incorporation of a common liquid electrolyte into a polymer, aiming at improving its properties. Besides, when the incorporation of fillers is accomplished, a new class of electrolytes arises that is titled “composite-polymer electrolytes” (CPEs).

**Figure 1:**
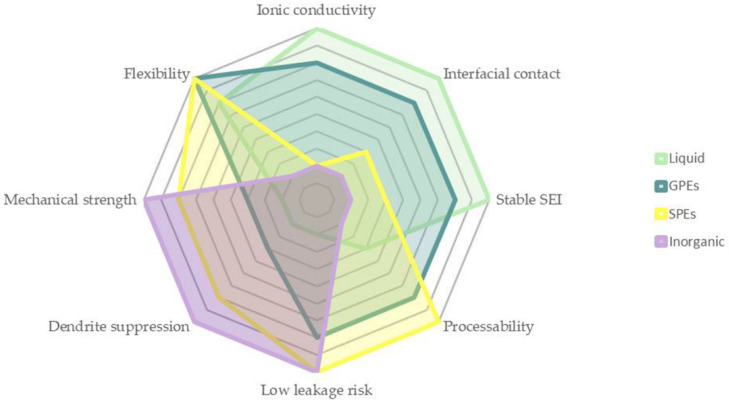
Some of the most important characteristics of electrolytes across different metrics.

Aside from the obvious morphological differences, the expected performance of each type of electrolyte is also noticeably distinct. Figure 1 summarizes the most important electrolyte characteristics for enhanced battery operation.

Liquid electrolytes are a mature technology that has shown to be reliable in terms of ionic conductivity, with values ranging from 10^−3^ to 10^−2^ S·cm^−1^ at room temperature (RT). Nevertheless, hazards (such as high flammability) and poor recyclability still restrict their use in a wide range of technological applications [8]. These limitations guide the development of novel and versatile high-performance electrolytes to significantly improve the safety issues and electrolyte leakages, which are known to limit the overall performance of a Li^+^-based battery [9,10,11]. This has led to a recent rise in promising alternative materials, including solid-state electrolytes due to their intrinsic slower reactivity, resulting in a longer device cycle life when compared to the liquid counterparts [12].

Inorganic electrolytes, although more ionically conductive, produce lower mechanical properties and poorer performance in highly demanding structural applications [13]. Conversely, organic electrolytes, such as carbon-based polymers, show greater flexibility and capacity of solvating ions, due to the presence of oxygen and nitrogen groups in their chemical structure. Polymer electrolytes are also normally cost-efficient compared to inorganic and liquid electrolytes. The major advantages of employing solid- or gel-polymer electrolytes result from the conjunction of three different dimensions of analysis: (i) mass-producibility [14], (ii) thermal conductivity and stability, and (iii) electrochemical stability, ionic conductivity > 10^−3^ S·cm^−1^ and, consequently, energy density. [15]. Unlike what occurs with liquid electrolytes, serial stacking of solid-state batteries becomes simpler/feasible. This can also improve the efficiency, reduce design problems, and increase the volumetric density of the overall system, Costs can also be reduced with the easier recovery of solid electrolytes and, as such, this dimension can also be taken into consideration.

The first attempts to produce polymer electrolytes (PEs) were done by Wright et al. [16], who investigated the ionic conductivity of doped poly(ethylene oxide) (PEO) with alkali metals in the mid-1970s [16,17,18]. In 1978, Armand et al. [19] studied the use of PEO in electrochemical applications, showing that the conducting character of this material is promoted through the amorphous regions by proposing a PEO-Li salt with an increased ionic conductivity (~10^−4^ S·cm^−1^) at 40–60 °C. Feuillade et al. [20] showed the conception of a quasi-solid GPE for a Li cell through the incorporation of plasticizers into a polymer-salt system. Another turning point in this field was accomplished by Skaarup et al. [21] in 1988, who developed a composite electrolyte. The authors created mixed-phase electrolytes based on lithium nitrile (Li_3_N) and lithium triflate (LICF_3_SO_3_) with PEO, resulting in an electrolyte with higher ionic conductivity. Some signs of progress were also achieved with special emphasis on inorganic (or ceramic) electrolytes. For example, Wieczorek et al. [22] added aluminum oxide (Al_2_O_3_) to enhance the ionic conductivity of PEO-based electrolytes, mainly promoted by an increase in the amorphous region.

Therefore, this review aims to show the main scientific progress in the design of versatile polymer-based electrolytes and composite electrolytes, underlining the current limitations and remaining challenges and highlighting their technical accomplishments. Special emphasis will be given to the potential employment of polymer electrolytes in structural batteries since they have been considered the most promising materials for these highly demanding applications.

## 2. Overview

**Figure 2:**
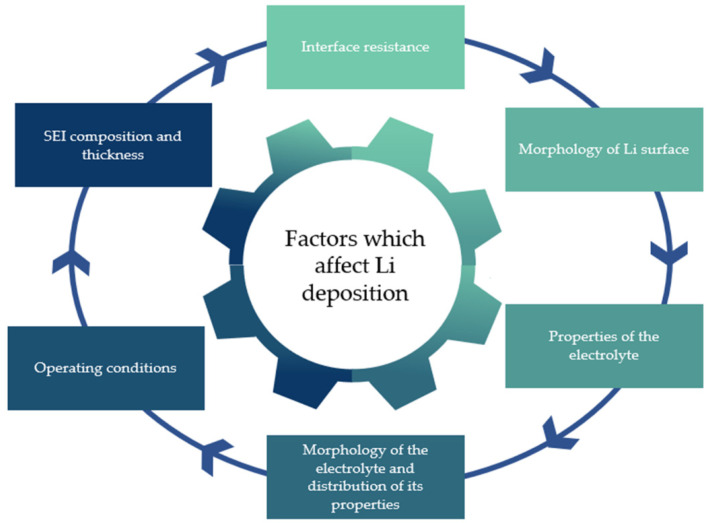
Additional factors that influence the Li deposition in a LIB cell.

Li-ion batteries already dominate the market, from laptops and smartphones to electric vehicles [23,24], because of their ability to enable high-energy- and high-power-density-demanding applications. LIBs are normally formed by a cathode, a separator, an anode, and an electrolyte. The latter has a crucial role in the proper operation of the supply system since it serves as a medium for Li-ion movement between both electrodes. The electrolyte must be a good insulator so that all electrons can be conducted exclusively by the external circuit. If the electrolyte is damaged, insulation and safety can become compromised and electricity production becomes impossible. Thus, the operating environment, abuse tolerance (such as overcharging), and battery chemistry [25,26,27] require special attention, as these determine the safety and performance of electrolytes, and ultimately the performance of LIBs. Important factors that influence the electrochemistry of batteries (Li deposition in a LIB cell) are presented in Figure 2.

Harsh conditions, high temperature, or mechanical damage [23,24,28,29,30,31] lead to increased risks of toxicity, leakage, and flammability in LIBs. In addition, the high reactivity between the electrodes and the electrolyte can promote the formation of an unstable solid-electrolyte interface (SEI) film [32,33]. This film contributes to an increase in the impedance, as the SEI layer is essentially formed by Li-based inorganic insulators, and a decrease in the electrical contact and capacity [34,35].

Lithium dendrite formation and growth, as well as parasitic reactions, are also a common drawback, and occur during the charge/discharge process, leading to the deposition of Li-rich compounds beyond metallic Li at the interface [7,36]. The consecutive deposition of this Li can promote short-circuits and can lead to electrolyte decomposition during cycling [37]. Other causes induce dendrite formation, including mechanically weak electrolytes, irreversible surface reactions, and unstable ion transport [38,39]. When combined, these promote complete failure, not only of the LIB but also of the device where the LIB is installed.

Strategies to overcome these safety challenges have been explored to optimize the overall performance of the electrolyte operation in a LIB cell. Alternative polymer electrolytes can produce innovations in mass savings, shape flexibility, and fire retardancy [40]. The pioneering work by Fenton et al. [17] showed that the ionic conductivities of modified polymers with inorganic salts, formed by complexation between PEO and alkali metals, stimulated substantial research into improved SPEs, new theoretical modelling of ionic transport, and a better understanding of the performance and properties of SPEs, such as the electrolyte/electrode interface [41,42]. Nonetheless, conductivities in the order of 10^−8^~10^−7^ S·cm^−1^, at RT, restrained the application of PEO-based SPEs.

Different methodologies have been investigated to improve this property, including the modification and optimization of the polymer backbone, polymer blending, and filler incorporation (polymer compounding) [43,44,45,46,47,48]. Undoubtedly, the application of an SPE in a LIB can produce high-energy, -density, -safety, and -power devices [49,50,51]. However, the interactions on the polymer–metal ion interactions are affected by a wide possibility of factors, including distance and composition between functional groups, the nature of the functional groups that are attached to the polymer backbone, molecular weight distribution, and the degree of branching [40,52].

Contrasting with this approach, in 1975, the concept of gel-polymer electrolytes was proposed by Feuillade et al. [7]. The properties of a GPE are an intermediate compromise between the features of an SPE and a liquid electrolyte. A GPE is obtained by incorporating liquid plasticizers and/or solvents into a polymer–salt system. The key functions of this plasticizer/solvent incorporation are to increase the content of amorphous regions in the electrolyte, to boost segmental motion, and to improve structural support, ultimately increasing the safety of usage by maintaining the GPE in a quasi-solid state [53,54]. Additionally, and unlike what happens in SPEs, Li-ion transport is not subjected to the segmental motion of the polymer; instead, the lithium ions move through both the gelled and liquid phases. In the first case, the Li^+^ transport happens when the membrane is homogeneous or when a low percentage of connected pores is formed. On the other hand, if the membrane is mainly constituted by connected pores, the conductivity will be mostly related to the properties of the liquid electrolyte. The most popular polymers used to produce GPEs include poly(ethylene oxide) (PEO), poly(vinylidene fluoride) (PVDF), poly(methyl methacrylate) (PMMA), polyacrylonitrile (PAN), and poly(vinylidene fluoride-hexafluoropropylene) (PVDF-HFP) because of their greater affinity with solvents and plasticizers that results from the polar nature of their bonds. In terms of benefits, GPEs have enhanced mechanical properties (in terms of flexibility, mechanical strength, etc.), high ionic conductivities, and superior electrolyte/electrode interfacial properties compared to liquid electrolytes. The biggest drawback of this type of electrolyte is the effect on ionic conductivity due to the added plasticizer. The addition of organic solvents creates unstable thermal behavior, which leads to events such as fire and explosions. Because of this, chemical and physical crosslinking, as well as physical support membranes, were introduced to overcome these shortcomings and improve mechanical strength. The incorporation of inorganic fillers has also helped improve the electrochemical and transport properties [5,7,13,55,56,57,58,59].

Considering what has been discussed so far, it is clear that this field requires optimization to not only diminish the safety hazards related to SPE/GPE employment, but also to enhance the overall performance of these types of electrolytes (and, consequently, the overall LIB performance). From Table 1, it is possible to compare some inherent properties of the electrolytes mentioned above.

As Table 1 shows, GPEs and SPEs exhibit better property balance than conventional liquid electrolytes. Polymeric materials, such as PEO/lithium perchlorate (LiClO_4_), PEO-tetraethylene glycol dimethacrylate (TEGDMA)—tetraethylene glycol dimethyl ether (TEDME)/lithium bis(trifluoromethanesulfonyl)imide (LiTFSI), PEO-sulfur-poly(ethylene glycol) methacrylate) (PEGMA)/LiTFSI, and polyethylene glycol (PEG)-hexamethylene diisocyanate trimer (HDIt)/LiTFSI were analyzed, considering the optimization strategy, ionic conductivity, electrochemical window of stability (EWS), and transference number (t_Li_+). Some recent approaches of polymers selected as promising electrolytes are outlined in Table 2, considering what was mentioned above.

The following question arises: What is expected from a polymer electrolyte to be usable in a LIB? There are fundamental properties that must be achieved and the combination of them in an electrolyte might be a challenge. It is expected that an ideal polymer electrolyte possess high ionic conductivity, nearly to the liquid electrolytes 10^−3^~10^−2^ S·cm^−1^, followed by a unity Li-ion transference number (t_Li_+), leading to a decrease in concentration gradients and preventing dendrite formation [5,45,64]. The latter two parameters have a direct impact on the performance of the cell, since the maximum power of the LIB is related to the conductivity and the maximum limiting current can be associated with t_Li_+. Good mechanical strength is foreseen to tolerate volume changes from the electrode constituents during the intercalation–deintercalation process. A low glass transition temperature (*T_g_*) will provide greater flexibility to the polymer chain, allowing ions to flow through the amorphous regions when the temperature is higher than *T_g_*. A wide electrochemical stability window desirably ≥ 4.5 V vs. Li/Li^+^ is also required, as it enables higher cathode material redox potentials [19]. Good contact at the electrode/electrolyte interface and excellent chemical stability is also required, as well as a low energy barrier for ionic conductivity between both components and high thermal stability for the safe operation of the LIB [42,55]. Moreover, the polymer shear modulus (G) should be at least twice the value of the Li metal to avoid penetration of Li sediments [37].

Considering the key challenges that must be overcome, it is evident that the complexity of the LIB optimization process lies in the compromise between the various performance metrics mentioned above. Therefore, a more intensive outlook on those parameters is depicted below.

## 3. Performance Metrics

The current limitations of SPEs/GPEs encompass several aspects, which need to be tuned during the polymer design, allowing the LIB cell to function at its finest. The core parameters to describe SPEs and GPEs are largely similar and are portrayed as follows.

### 3.1. High Ionic Conductivity

**Figure 3:**
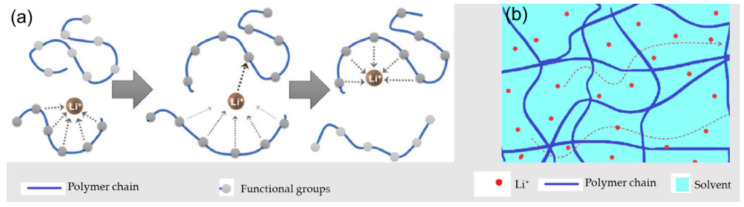
Representative scheme of (**a**) the predominant Li-ion transport in SPEs and (**b**) GPEs. Adapted with permission from Wu et al. [14]. Copyright 2021 Elsevier.

The employed electrolyte must act as an electrical insulator and ionic conductor, as highlighted before, serving as the dominating parameter for polymer electrolytes [7,65]. To have practical application, this specification should achieve the ionic conductivities of liquid electrolytes, which typically reach values of 10^−3^~10^−2^ S·cm^−1^, at RT, as mentioned previously. The internal impedance and the electrochemical behavior are severely affected by ionic conductivity. In Figure 3, a representative scheme on the differences between ion transportation in SPEs and GPEs is presented.

For dry solid-polymer electrolytes, it is accepted that ion conduction does not occur in the well-organized phase. In SPEs, and looking at Figure 3a, the ion conduction mechanisms involve both salt dissociation and complexation with the functional groups in the polymeric chain, as well as hopping off the Li^+^-ions between the coordinated sites [13,66]. For high-molecular-weight polymers, the main form of ionic conduction is intersegmental hopping, whereas for low molecular weights, the ionic conduction occurs mostly through diffusion [38]. Regarding GPEs, and considering their composition, both Li salt and solvent selection are preponderant, as well as the salt’s concentration. In this case, the contributions of those parts are not easy to describe because the morphology and the polymer–solvent interactions are complex.

Several models have been developed to describe ionic conductivity (*σ*), although the most accepted in the scientific community for a homogeneous media (liquid electrolytes and amorphous polymers) is Vogele–Tammane–Fulcher (VTF) [40], represented in Equation (1):

(1) $$\sigma \;\left(T\right)=AT^{-\frac{1}{2}}\exp\left[-\frac{B}{K_{B}\left(T-T_{0}\right)}\right]\;$$

where *T* is the absolute temperature, A is the pre-exponential factor related to the number of charge ions, $K_{B\;}$ is the Boltzmann constant, *T*_0_ (*T*_0_ *= T_g_* − 50 *K)* is the critical temperature at which configuration entropy or free volume disappears, and B is the pseudo-activation energy related to the segmental movement [13]. The VTF model correlates the conductivity with the segmental relaxation in polymers and indicates that the motion of the polymer chain increases when the temperature or the free volume available increase [67]. Consequently, the freer the volume available, the more interchain hopping and interchain ion movement that happens, leading to an increase in the amorphous state of the polymer and the ionic conductivity.

**Figure 4:**
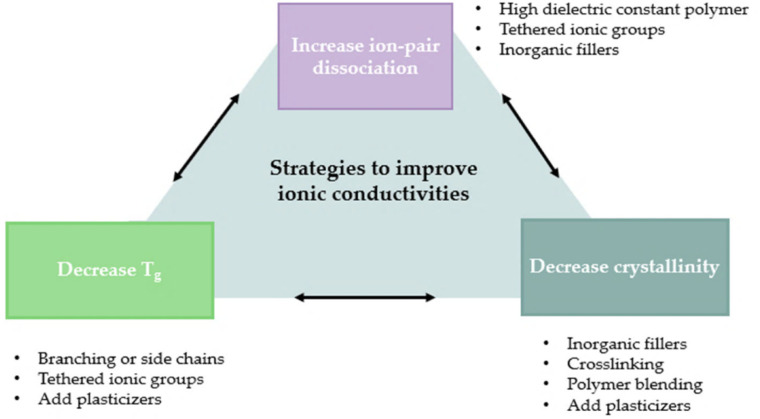
Summary of the principal strategies to improve ionic conductivities of polymers in SPEs and GPEs [68].

However, sometimes the preferable path for ionic motion is jumping from another site of complexation, and, in this case, the ionic conductivity can be expressed by the Arrhenius equation, as represented in Equation (2):

(2) $$\sigma =\sigma_{0}\exp\left(\frac{-Ea}{K_{B}T}\right)\;$$

where$\;\sigma_{0}$ is the pre-exponential factor, *Ea* is the energy activation, and *T* is the temperature. Equations (1) and (2) allow a better understanding of the key points to increase ionic conductivity. Although both mechanisms are correct, their predominance depends on the structure, temperature, and constituents of the system. Therefore, in both SPEs/GPEs, there are several aspects and strategies in common to improve the ionic conductivity of the employed polymer, which are briefly summarized in Figure 4.

### 3.2. Decrease Glass Transition Temperature and Crystallinity

**Figure 5:**
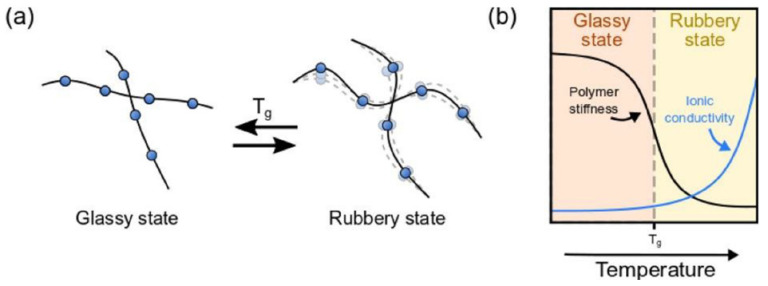
(**a**) Representative scheme of the behavior of the polymer related to the glass transition temperature and (**b**) interpretation of the relationship between the ionic conductivity and the polymer stiffness. Reprinted with permission from Gebert et al. [13]. Copyright 2021 Elsevier.

The ionic conductivity is highly affected by the glass transition temperature and crystallinity of the polymer matrix. Above this temperature, Li^+^ ions can either hop from one chain to another segment motion or relocate from one coordination site to a new one or even induce a shift in the free volume of the matrix [13]. Usually, polymers exhibit an insignificant ionic conductivity below *T_g_*, and, therefore, the desire is to decrease *T_g_*, aiming to obtain a rubbery polymer at RT. Based on this, the main approaches for decreasing *T_g_* are through the incorporation of plasticizers, solvents, ionic groups, and/or side chains. Figure 5 shows a representative scheme of how *T_g_* affects ionic conductivity.

Plasticizers, with a low molecular weight, organic solvents, or ionic liquids, can increase the amorphous content of a polymer and, thus, increase the ionic conductivity [11]. For example, low-molecular-weight polyethylene glycol (PEG) has been broadly applied as a plasticizer in PEO–salt complexes [7]. Research conducted on this subject shows that the incorporation of PEG into a PEO-LiCF_3_SO_3_ system leads to a conductivity improvement from 10^−7^ to 10^−4^ S·cm^−1^ at 40 °C. Moreover, Cao et al. [69] developed an electrolyte with the composition (PEO)15/LiTFSI/10 wt.% polyethylene glycol dimethyl ether (PEGDME), which enabled an ionic conductivity of 10^−3^ S·cm^−1^ at 50 °C. At 27 °C, a conductivity of 1.60 × 10^−4^ S·cm^−1^ was found by Johan et al. [70] by preparing a polysiloxane (PSi)/15 wt.% LiCF_3_SO_3_/40 wt.% borate ester B_3_. The use of organic solvents helps solvate ions and facilitates their transportation. A high dielectric constant and low-viscosity solvents are required, although others such as dichloromethane (DCM) have also proven to be a reliable choice. Yu et al. [7] showed that a novel system based on synthesized poly(propylene carbonate maleate) (PPCMA)/1.0 M LiClO_4_/ethylene carbonate + dimethyl carbonate (EC + DMC) (1:1, vol.%) can exhibit a similar conductivity (8.43 × 10^−3^ S·cm^−1^, at RT). Lastly, ionic liquids are temperature molten salts with unique properties, and several of them have been investigated as potential plasticizers, considering a combination of cations such as pyridinium ([pyr]^+^), imidazolium ([EMIM]^+^), piperidinium ([BMPip]^+^), quaternary ammonium ([NR_4_]^+^) with anions as hexafluorophosphate ([PF_6_]^−^), tetrafluoroborate ([BF_4_]^−^), dicyanimide ([N(CN)_2_]^−^), and bis[(trifluoro methyl)sulfonyl]imide ([CF_3_CONCF_3_SO_2_]^−^), among others.

The ionic conductivity is also greatly influenced by the polymer crystallinity of the electrolyte, considering that the Li^+^ transport is typically correlated to the motion of the polymer chain segments and mainly occurs in the amorphous phase of the SPE. Some strategies have been studied and reported in the literature to decrease crystallinity. As mentioned before, both plasticizers and inorganic fillers can increase the amorphous content. On the other hand, the reduction of Li^+^ diffusion pathways allied with the enhancement of the cell capacity makes the idea of the incorporation of the inorganic filler very attractive [33].

For example, inert oxide fillers enhance the electrochemical properties by decreasing the crystallinity of the polymer and boosting the establishment of channels by the Lewis acid–base interaction between the chains and fillers [71]. Silicon oxide (SiO_2_) has been considered the “holy grail” due to its unique characteristics. Huang et al. [72] stated that SiO_2_ fillers improved the stability and the chemical behavior of PPC-based electrolytes. Furthermore, SiO_2_ can also act simultaneously as a crosslinking agent, according to Zhu et al. [73]. Aluminum oxide (Al_2_O_3_), zirconium dioxide (ZrO_2_), lithium aluminum oxide (LiAlO_2_), and titanium oxide (TiO_2_) have also been investigated as promising fillers to enhance ionic conductivity.

**Figure 6:**
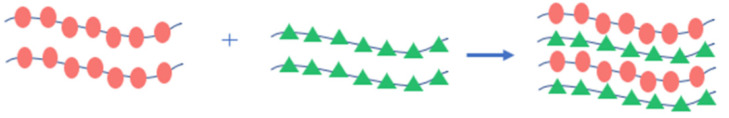
Representative example of a blending polymer.

Other strategies have been investigated to target the decrease in crystallinity of SPE matrix, namely, polymer blending and crosslinking. These strategies will be discussed in detail in topic 4, although they will briefly be presented. Blending implies the mixture of two or more types of polymer chains without chemical bonding between them. This process demolishes the consistency of a single polymer chain, forming the desired amorphous polymer. For example, according to Tanaka et al. [11], the blending of PEO with poly(ethylene imine) (PEI) resulted in an ionic conductivity of ~10^−4^ S·cm^−1^ at 30 °C. A representative example of a blending polymer is presented in Figure 6.

Crosslinking also induces a decrease in crystallinity by obtaining an amorphous polymer without compromising its mechanical properties and improving its chemical and electrochemical stability. The crosslinking reactions between end groups ensure that polymer chains are locked up, preventing interchain crystallization [38]. The first report of this reaction for dendrite growth suppression was based on the incorporation of polyethylene (PE) in a PEO, resulting in an electrolyte with improved ionic conductivity (~10^−5^ S·cm^−1^ at RT) [74].

### 3.3. Increase Ion-Pair Dissociation

The dissociation of salts is possible with the employment of high dielectric polymers, tethered ionic groups, and/or inorganic fillers. For instance, for PEO-based electrolytes, the dissociation of salts is not fully accomplished due to their low dielectric constant, resulting in ion aggregation and, consequently, lower ionic conductivities [5]. Aiming at ensuring an adequate dissociation of Li salts, the polymer should contain polar groups and the formation of complexes in its molecular chain [75]. However, when those functional groups are attached to the polymer backbone, a more efficient electrolyte is attained in terms of ionic conduction and electrochemical stability. Anion receptors are also a strategy to improve the movement of Li ions, considering that the anion mobility can be hindered by the strong ion–dipole interaction between anions and receptors, enhancing the conductivity [76].

### 3.4. Lithium Transference Number

Along with high ionic conductivity, an electrolyte with a close-to-unity Li^+^ transference number (t_Li_+) is a desired requirement, as it can produce higher power density during the charge and discharge process, preventing the formation of dendrites [7].

To calculate t_Li_+ the Bruce-Vincent equation is commonly used, along with an electrochemical test that involves the polarization of a cell to induce a concentration gradient and reach the steady state [18], according to Equation (3):

(3) $$t_{Li}+=\frac{I_{s}\left(\Delta V-I_{0}R_{0}\right)}{I_{0}\left(\Delta V-I_{s}R_{s}\right)}\;$$

where *I_S_* and *I*_0_ are the steady-state and initial currents, respectively; Δ*V* is the applied potential; and *R_s_* and *R*_0_ are the steady-state and the initial interfacial resistances, respectively.

This parameter is indirectly related to the total ionic conductivity provided by Li^+^ [77]. The Li deposition, which is responsible for dendrite formation, is promoted by the simultaneous movement of Li ions and anions, but in different directions, leading to an increase in the Li-ion gradient between the anode and the cathode. The usual low t_Li_+ is due to the small volume of Li^+^ and, if the transference number is too small, to an accumulation of anions at the electrode surface [75].

There are two main approaches to reducing anion mobility: (i) The first is through the introduction of anion receptors into the electrolyte system to interact with the anion, and (ii) the second comprises the attachment of anions to the polymer backbone, allowing the formation of single-ion polymer electrolytes. Those can act as SPEs or, if plasticized with a salt-free electrolyte, as GPEs [13].

### 3.5. Electrochemical Window of Stability

The requested operating voltage defines the necessary electrochemical window of stability (EWS), which can be defined as the difference between the potential of oxidation and the potential of reduction [7]. The first requirement is that the electrolyte be inert to both electrodes. The oxidation potential should be higher than the potential of Li^+^ in the cathode and the reduction potential should be lower than that of lithium metal in the anode in the absolute potential scale [77]. This is one of the most important parameters to consider since it reflects not only the electrodes’ reactivity but also the stability of the system in a certain operating voltage. Ideally, polymer electrolytes should have an EWS of at least 4–5 vs. Li/Li^+^ to be compatible with traditional pairs of electrode materials with high-voltage cycling plateaus, such as lithium nickel manganese oxide (LNMO) and nickel manganese cobalt oxide (NMC). The EWS can be evaluated from linear sweep voltammetry or even cyclic voltammetry.

### 3.6. Mechanical Stability and Shear Modulus

Mechanical performance and stability are crucial parameters for polymer electrolyte applications. The electrolyte must not be brittle and should be flexible, elastic, and be able to stand the stress conditions during the cell package/usage [7,77]. Moreover, the electrolyte should be able to endure emergencies during the cell lifetime and high temperature, and even suppress dendrite growth [78]. To increase the mechanical stability, approaches such as adding inorganic fillers, crosslinking, blending with high-strength polymers, and introducing rigid blocks have been reported [78]. The shear modulus (G) is another important factor to avoid Li deposition, and according to the literature, G should assume values twice as high as those of Li metal, suggesting a G > 6 GPa [37].

### 3.7. Chemical and Thermal Stability Range

During the operation of the battery, the chemical stability of the electrolyte plays an important role and should prevent the occurrence of adverse chemical reactions, and the thermal stability must ensure the safety of the electrolyte itself [33]. Additionally, electrolytes must be inert and avoid damage to the remaining battery constituents, including cathodes, anodes, current collectors, cell separators, and cell packaging materials [77].

## 4. Design of Solid- and Gel-Polymer Electrolytes

To modulate the desired properties and performance of polymeric electrolytes, numerous studies have been carried out, namely, on the development of traditional polymeric blends, as well as the design and synthesis of new polymers, with improved properties and unique structures. Electrochemical properties for recent promising studies are reviewed in this section and are summarized in Table 3.

### 4.1. Novel and Versatile Polymer Electrolytes with Advanced Properties

The well-known limitations of traditional commercial polymer matrices represent a major drawback for SPE employment. The current state of the art is mainly focused on the optimization of PEO-based electrolytes or other traditional polymeric matrices; however, the need to develop new synthetic strategies for the discovery of innovative monomers and polymers is crucial to achieving future energy storage applications with tuned mechanical and electrochemical properties. Through organic reactions, such as reversible addition−fragmentation chain-transfer polymerization (RAFT), atom transfer radical polymerization (ATRP), sulfonations, thiol-ene chemistry, acid–base reactions, anionic exchange, and amine–epoxy couplings, novel polymers can be designed by simple approaches and at low cost. Afterwards, those can be characterized by standard analytical techniques, namely Fourier-transform infrared spectroscopy (FTIR), nuclear magnetic resonance (NMR), liquid chromatography–mass spectrometry (LC-MS), gas-phase chromatography (GPC), differential scanning calorimetry (DSC), etc. Several synthetic strategies have been proposed to modulate the polymeric backbone of the SPE, regarding the decrease of crystallinity, improved mechanical performance or high t_Li_+, and conductivity. Moreover, dendrite suppression and higher ionic conductivity at RT are also taken into consideration. The most promising recent works comprising the modification of the polymer chain, copolymerization, or crosslinking, as well as the use of highly delocalized moieties, are subsequently reviewed in terms of their synthesis, design, and assembled cell performance.

#### 4.1.1. Polymer Matrices and Blending

PEO, poly(vinylidene fluoride) (PVDF), and poly(acrylonitrile) (PAN), are the most common polymer matrices studied for SPE development. However, it is stated in the literature that these single polymers cannot fulfil all the requirements for developing advanced and structural batteries. As mentioned before, a simple and cost-effective method for obtaining solid electrolytes can be achieved by blending two or more versatile polymers with Li^+^ salt in the presence of a solvent that can properly dissolve all materials. After solvent removal, the resultant membranes can benefit from the combined properties of single polymers, namely, mechanical strength and thermal stability, or ionic conductivity and other electrochemical properties.

Putri et al. [79] recently prepared a PEO/poly(vinyl alcohol) (PVA) blend with lithium hydroxide (LiOH) as a source of Li^+^ cations. The addition of amorphous PVA was determinant to disrupt PEO crystallinity and enhance the SPE conductivity at RT. The researchers applied ultrasonication to improve the conventional solution casting method by enhancing the miscibility of the components in the precursor solvent, resulting in a more homogeneous solution in a relatively shorter time. It was found that a concentration of 2 wt.% of LiOH was sufficient to decrease the crystallinity phases of the blended polymers, leading to superior conductivity (1.25 × 10^−4^ S·cm^−1^, 25 °C) in comparison with traditional single PEO membranes, which only displayed RT ionic conductivities in the order of 10^−6^ S·cm^−1^. Moreover, PEO/PVA/LiOH (2 wt.%) membrane showed wide electrochemical stability up to 5 V (vs. Li/Li^+^), suggesting that it can be applied as a solid polymer electrolyte for battery technologies.

The use of PVDF-HFP-based electrolytes is a decent alternative to the use of PEO due to its safety and flexibility as a polymer; however, the lack of ionic conductivity and poor mechanical integrity still represent a major drawback for practical applications. In a recent study, PVDF-HFP was blended with an ultraviolet (UV) curable pentaerythritol tetracrylate (PETEA), a semi-interpenetrating polymer capable of forming a mechanically sound network with conductive capability [80]. In fact, after swelling in proper plasticizer, the resultant PVDF-HFP/PETEA GPE (4:1 ratio) not only showed good mechanical properties, such as flexibility and foldability, but also presented improved thermal safety, as well as high RT ionic conductivity (5 × 10^−4^ S·cm^−1^), which was about 2.5 times higher than the single PVDF-HFP membranes. The addition of UV-induced polymerizable moiety resulted, as expected, in better interface compatibility between the electrolyte and the electrodes, crucial to enhancing the batteries’ safety and performance. Consequently, the assembled cell exhibited a wide electrochemical window of 4.8 V (against Li/Li^+^), as well as an initial discharge capacity of 151 mAh·g^−1^ at a 0.5 C rate and decent 98% cycling retention after 50 cycles, suggesting that blending could provide a simple, cost-effective method to develop future polymeric batteries.

Inspired by the necessity to develop new alternatives for the construction of safer batteries, Mocek et al. [81] developed a versatile PVDF-based GPE that used lithium difluoro(oxalate)borate (LiODFB) and lithium bis(oxalate)borate (LiBOB) salt, in replacement of the toxic and highly reactive LiFP_6_ salt currently used for battery production. GPE membranes were prepared by the solvent-casting method, comprising PVDF with a combination of borate-based Li salt at different ratios and ionic liquid (EtMeImNTf_2_ or MePrPyrNTf_2_) and sulfolane (TMS) as plasticizers. Comparably with traditional Li^+^ sources, borate-based Li^+^ salts also improve thermal stability, have a wide electrochemical window (up to 5.6 V), and have the capacity to form a stable SEI layer that ensures good cycling stability and safety for the battery [91].

On the other hand, ionic liquids have been appointed as a greener alternative for traditional plasticizers due to their non-flammability, suitable ionic conductivity, and superior thermal, chemical, and electrochemical stability, thus providing superior safety for future batteries [92]. As a result, the developed free-standing, flexible, and highly conductive thin films (3.21 × 10^−3^ S·cm^−1^, 25 °C) did not release flammable products when subject to flame tests. However, due to the low solubility of the LiODFB lithium salts in the plasticizer, the resultant transference number was only 0.03, which is not convenient for practical applications.

Ionic liquids serving as a plasticizer is another route where a polymer blend was developed, which also contained grafted nanoparticles for enhanced performance. Particularly, different poly(acrylonitrile-polyhedral oligomeric silsesquioxane)/PVDF (PAN-POSS/PVDF) GPE was prepared, with the incorporation of ionic liquid as a plasticizer and POSS nanoparticles that were grafted to PAN chains [82]. The incorporation of PAN favored not only the compatibility with the electrode but also the dendrite suppression, which could not be reached by PVDF itself. This behavior can be explained considering that PAN, containing electron-withdrawing and polar nitrile groups, exhibits good thermal and mechanical properties, as well as outstanding electrochemical performance [93]. However, increasing the content of PAN-POSS in the PVDF matrix led to a higher pore content in the membrane structure, which consequently absorbed the more ionic liquid and limited its mechanical robustness. Nonetheless, the incorporation of POSS nanoparticles enabled further improvement of the thermal, mechanical, and electrochemical properties of the electrolyte membrane, as reported in previous studies [94,95,96]. Results showed that 15 wt.% PAN-POSS/PVDF-based GPE exhibited superior RT ionic conductivity, as high as 1.91 × 10^−3^ S·cm^−1^. When assembled in a Li/LiFePO_4_ cell, it displayed a wide electrochemical window of 4.6 V and an initial discharge capacity of 112 mAh·g^−1^ at a 0.1 C rate.

#### 4.1.2. Copolymers

The use of copolymers represents a captivating alternative to obtain a set of desired properties. For instance, the combination of monomers that allow a high ionic conductivity with others containing more rigid domains will allow the formation of hybrid systems with different optimized ionic and mechanical properties, which generally do not coexist in traditional matrices. Another advantage of using these types of materials is the possibility of disrupting the crystalline phase, typically present in traditional PEO electrolytes, which allow ionic movement. Block copolymers and graft copolymers have been the most studied copolymer type for SPE and GPE development.

The first attempts conducted by Giles et al. [97,98] in 1987 demonstrated the development of graft copolymers of styrene–butadiene–styrene (PS-P(B-*g*-MPEG)-PS) with PEO of chains of different lengths grafted to the ABA triblock copolymer. The subsequent mixture with different LiCF_3_SO_3_ salt ratios resulted in the desired polymer electrolytes, which exhibited an amorphous character at different temperature conditions. Decent ionic conductivity of 10^−5^ S·cm^−1^ at RT was achieved.

**Figure 7:**
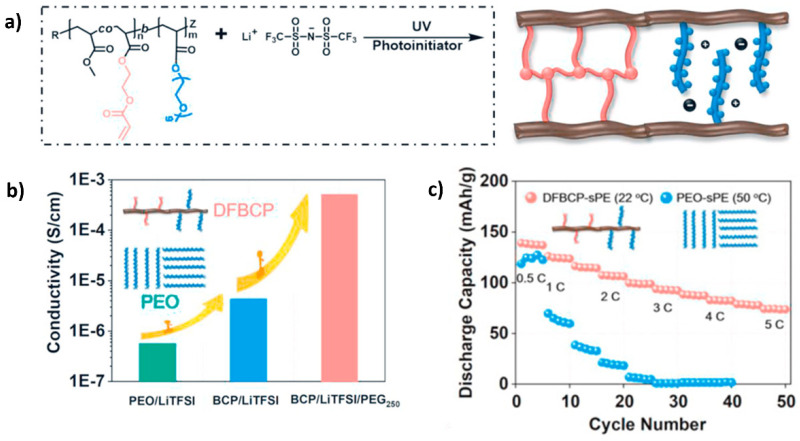
(**a**) Schematic representation of the block copolymer SPE prepared by UV polymerization with LiTFSI salt; (**b**) comparison of experimental ionic conductivity of the developed membrane with reported PEO and BCP membranes; (**c**) cycling performance of Li/DFBCP-sPE/LFP cell at 22 °C and Li/PEO-sPE/LFP cell at 50 °C. Reprinted with permission from He et al. [83]. Copyright 2021 Elsevier.

He et al. [83] prepared an SPE consisting of a difunctional block copolymer (DFBCP) prepared by sequential RAFT polymerization. Firstly, a polymer block containing acrylate moieties P(HOEA-co-MA) was synthesized by a reaction of methyl acrylate (MA) and 2-hydroxyethyl acrylate (HOEA). Subsequently, polymerization with PEG monomers followed by reaction with acryloyl chloride provided the desired DFBCP containing terminal cross-linkable vinyl bonds and ion solvation groups in separated blocks of the polymer. The final SPE was prepared by UV crosslinking with LiTFSI salt (Figure 7a) and showed improved ionic conductivity compared to traditional linear PEO or another block copolymer (BCP) SPEs (Figure 7b). This resulted from the mobile PEG chains, which allow faster ion solvation and a lower degree of crystallization, which were not confined by the crosslinking network due to being in different blocks of the copolymer. Enhanced mechanical performance was also achieved by the presence of the crosslinking sites.

Copolymerization proved to be efficient to combine mechanical performance without compromising conductivity via the combination of different di-block polymer architectures. After assembling, the Li/LiFePO_4_ cell exhibited an initial capacity of 101.4 mAh·g^−1^ (2 C rate, 22 °C, Figure 7c) and impressive long-term cycling stability up to 1000 cycles. At a higher current of a rate of 4 C, the cell also displayed a decent capacity of more than 70 mAh·g^−1^ for over 200 cycles.

**Figure 8:**
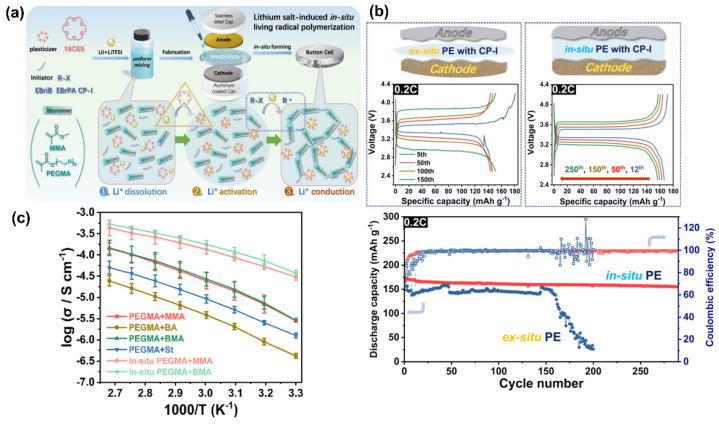
(**a**) Schematic illustration of in-situ polymerization via Li^+^ salt-induced radical polymerization; (**b**) charge–discharge curves at different cycle numbers of the Li/LiFePO_4_ cell via (1) ex-situ or (2) in-situ assembling with P(PEGMA-co-MMA)-based PE, initiator (CP-I), and (3) cycling performance of the Li/PE/LiFePO_4_ cell conducted at 60 °C; (**c**) ionic conductivity of developed PEs at different temperatures. Adapted with permission from Yu et al. [84]. Copyright 2021 American Chemical Society.

Moreover, in-situ radical polymerization is another simple technique capable of enhancing the interface between electrodes and electrolyte, which is essential to improve the battery’s final performance, safety, and stability [99,100,101,102]. Moreover, it can simplify the use of traditional solvent-casting techniques by being a greener and faster alternative that does not overuse volatile solvents. Yu et al. [84] successfully developed an SPE by Li salt-induced copolymerization of poly(ethylene glycol) methacrylate (PEGMA) and several acrylate monomers using LiI and LiTFSI salts as activators and Li-ion sources. 18-crown-6-ether (18C6) served as both copolymerization solvent and plasticizer, and ethyl α-bromophenylacetate (EBrPA) or 2-iodo-2-methylpropionitrile (CP-I) were used as initiators (Figure 8a). This study suggested that using in-situ Li salt-induced polymerization could effectively contribute to uniform interface formation, as well as stable ion solvation, due to the substitution of traditional catalysts capable of accumulating and reacting with the electrodes surface for common Li salts that trigger an activation effect over typical polymerization initiators.

The fabricated Li/LiFePO_4_ cell with P(PEGMA-co-MMA)-based electrolytes exhibited not only good electrochemical stability of up to 5.20 V (vs. Li^+^/Li) but also an initial discharge capacity of 166.5 mAh·g^−1^ at 0.2 C and superior cycling performance (Figure 8b) compared to the traditional ex-situ systems, as well as subsequent dendrite suppression, even after 290 cycles. However, the lack of high ionic conductivity at RT (up to 10^−6^ to 10^−5^ S·cm^−1^) and low transference number (0.37) are the disadvantages of using this type of SPE, as shown in Figure 8c.

Inspired by previous studies containing poly(ε-caprolactone) (PCL) as a polymeric matrix for SPE development, Zhang et al. [85] designed a BAB triblock copolymer containing two monomers of PCL and one monomer of poly(propylene carbonate) (PPC). This strategy successfully improved SPE’s ionic conductivity at RT by introducing amorphous regions, due to PPC block, that provided higher ionic conductivity (3 × 10^−5^ S·cm^−1^) compared to the use of single semi-crystalline PCL. The resultant Li/LiFePO_4_ cell, containing SPE membrane with an optimal 20 wt.% of LiTFSI salt and triblock copolymer, displayed a wide electrochemical window of 5 V and was able to deliver a discharge capacity of 142 mAh·g^−1^ (0.05 C, RT), with 90% of the retaining capacity after 200 cycles. Furthermore, no dendrite formation was observed after long-term cycling, which demonstrates that this copolymer can successfully provide both safety and high energy density to future cell development.

**Figure 9:**
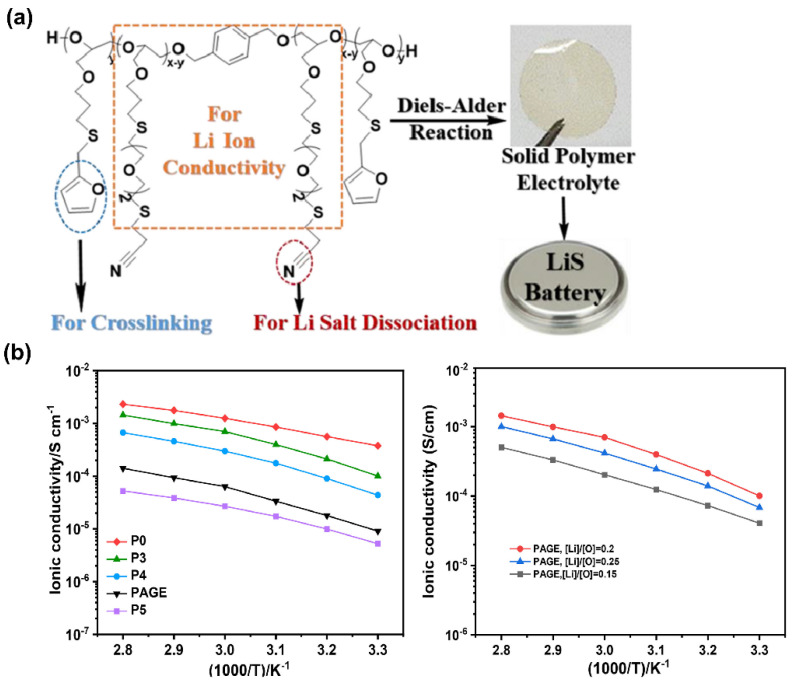
Schematic illustration of (**a**) structure of CN/FM-PAGE-based polymer with crosslinking sites for SPE formation via Diels–Alder reaction; (**b**) dependence of ionic conductivity with temperature by variation of mol% of crosslinking with Li/O = 0.2 (left) and variation of Li/O ratio (right). Reprinted with permission from Mallela et al. [86] Copyright 2020 Elsevier.

Moreover, new crosslinked structures based on poly(allyl glycidyl ether) PAGE copolymers with pendant nitrile (CN) and furfuryl mercaptan (FM) groups were developed to form an SPE with crosslinking sites via Diels–Alder reaction between the FM moieties in the presence of bismaleimide (Figure 9a) [86]. The highest value of 1.01 × 10^−4^ S·cm^−1^ of ionic conductivity at RT was attributed to the presence of CN groups, responsible for an effective Li^+^ dissociation, as well as the existence of a polyether backbone for Li^+^ migration. Different crosslinking ratios were studied to find the best compromise between ionic conductivity and mechanical strength, as crosslinking is known to increase the stiffness of a polymer. Results showed that the developed SPE with a 3% degree of crosslinking and a Li/O ratio of 0.2 showed superior ionic conductivity (Figure 9b), which inevitably decreased with the increasing number of crosslinking sites or Li–salt concentration, due to suppression of polymer chain mobility and the formation of aggregated species, respectively.

#### 4.1.3. Crosslinking

As mentioned earlier in this review, another approach applied to SPE development consists of using crosslinked structures by providing enhancement of mechanical properties through permanent covalent bonds formed between two types of monomers. Moreover, crosslinking is known to reduce the crystalline domains of a polymer, resulting in enhanced conductivity, as the amorphous regions enable higher ionic conductivity [33]. The possibility of combining monomers with different structure–property relations can result in novel and versatile membranes with combined ionic, mechanical, or safety-related properties. Inspired by novel research in the application of polymeric ionic liquids (PILs) for the development of safer polymer electrolytes [103], Tseng et al. [87] recently proposed a highly crosslinked membrane that incorporated a vinyl dicationic imidazolium ionic liquid (XVIm-TFSI) as a crosslinker, which was compared to a PIL-based copolymer without crosslinking units. This works relied on the capacity of PILs to display good capacity for ion solvation, due to their weak electrostatic interactions with cations, improving the safety since they are thermally stable structures [104]. In-situ polymerization, under solvent-free conditions, provided a highly crosslinked membrane that showed good RT ionic conductivity higher than 10^−4^ S·cm^−1^ and a wide electrochemical window up to 5.4 V (against Li/Li^+^). This optimized membrane (XP-20) contained a mixture of poly(ethylene glycol) methyl ether methacrylate (PEGMEA), LiTFSI, and poly(ethylene glycol) dimethyl ether (PEGDME) at a 5:3:4 weight ratio and a 20 wt.% ratio of the XVIm-TFSI crosslinker without the addition of plasticizers. As shown, the effect of the imidazolium crosslinker proved to enhance not only the ionic conductivity of the membranes but also the compressive elastic modulus, as well as its thermal stability, compared to the PIL-based copolymer, without crosslinking sites (P-20).

In addition, crosslinking successfully improved the membrane cycling stability against dendrite formation due to reduced polarization, stable SEI formation, and superior mechanical strength. The Li/LiFePO_4_ cell naturally showed a good specific capacity of 160 mAh·g^−1^ at a 0.2 C rate while maintaining 93.8% capacity retention after 150 cycles, a characteristic that was not present in the copolymer without the imidazolium crosslinker, where the performance of the cell continuously faded over cycling.

**Figure 10:**
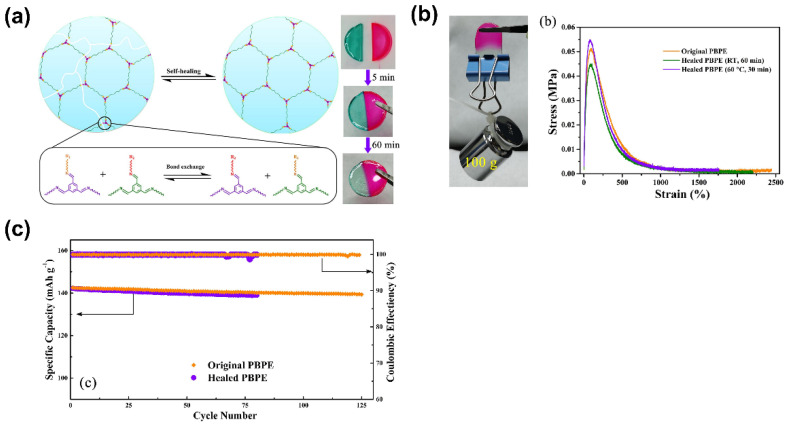
(**a**) Schematic representation of the self-healing electrolyte and photograph of the self-healing test; (**b**) photograph of the polymer membrane bearing a weight of 100 g and stress–strain curves of the original and healed membranes; (**c**) cycle performance of the original and healed PBPE electrolyte’s respective LFP/Li cells at a 1C rate. Reprinted with permission from Deng et al. [88] Copyright 2022 Elsevier.

Regarding the safety and stability of the battery, self-healing materials can provide an effective alternative to deal with damage related to mechanical loads. Deng et al. [88] recently proposed a self-healing polymer based on cross-linkable poly(ethylene glycol) diamine (NH_2_-PEG-NH_2_) and benzene-1,3,5-tricarbaldehyde (BTA) dynamic imine bonds (Figure 10a). These reversible covalent bonds proved to be capable of healing within 1 h while retaining their stability. Interestingly, even with the addition of a plasticizer, the healed GPE was able to bear a weight of 100 g without tearing (Figure 10b left). Moreover, stress–strain experiments showed a recovery of almost all its mechanical properties after healing (Figure 10b right), which demonstrates an advantage of using this type of electrolyte in future applications. Along with these important properties, the developed GPE showed a superior ionic conductivity of 4.79 × 10^−3^ S·cm^−1^ at 30 °C (Figure 10c left), which are good mechanical properties. The assembled LPF cell displayed an excellent specific capacity of 118.2 mAh·g^−1^ (5 C rate) and 97.8% capacity retention after 125 cycles (Figure 10c).

**Figure 11:**
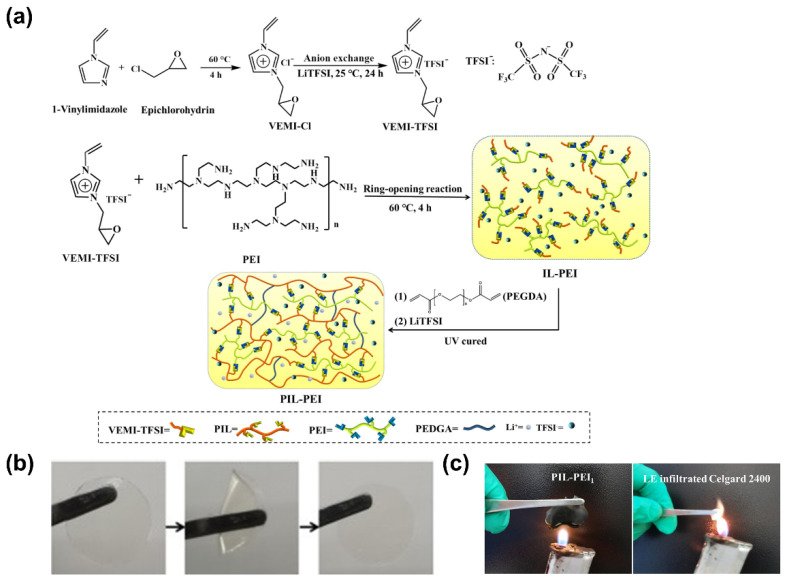
(**a**) Synthetic strategy to prepare the crosslinked PIL-PEI; (**b**) photograph of the developed membrane while being folded; (**c**) membrane flame test: PIL-PEI (left) and commercial Celgard (right). Reprinted with permission from Liang et al. [89] Copyright 2021 Elsevier.

Epoxy resins are interesting systems that can provide a high degree of crosslinking and mechanical strength. Their chemical nature enables polymerization upon heating without needing thermal radical initiators, known to produce by-products that are highly Li-metal-reactive species, covering the surface of the Li metal and thus increasing the electrode resistance and severely degrading the battery performance and stability. Moreover, these systems can be used to produce polymer electrolytes by simple production methods that promote the formation of stable electrode–electrolyte interfaces [105]. Relevant approaches based on epoxy systems were reported, where high ionic conductivity of around 10^−5^ to 10^−3^ S·cm^−1^ was successfully achieved [105,106,107,108]. Liang et al. [89] recently developed a GPE epoxy resin based on PEI and non-flammable ionic liquid monomer that also contained vinyl groups for dual crosslinking. The strategy of synthesizing a new type of bifunctional ionic liquid (Figure 11a) provides superior safety regarding the non-flammability of ionic liquids, without compromising ionic conductivity of the final system, as they replace the need for flammable plasticizers. After thermal and UV polymerization, the resultant flexible GPE presented excellent thermal stability (360 °C) (Figure 11b,c) and fair mechanical strength, while providing excellent ionic conductivity of 1.03 × 10^−3^ S·cm^−1^ at RT and fair t_Li+_ of 0.47. Inhibition of dendrites was also observed, owing to the formation of a stable SEI layer. The assembled Li/LiFePO_4_ cell showed a high specific capacity of 165.6 mAh·g^−1^ and stability for over 200 cycles, making this an interesting approach to future flexible device applications. This study demonstrated that ionic liquids and polymers can be applied to batteries, creating a new class of conductive electrolyte membranes that do not require the use of flammable plasticizers.

**Figure 12:**
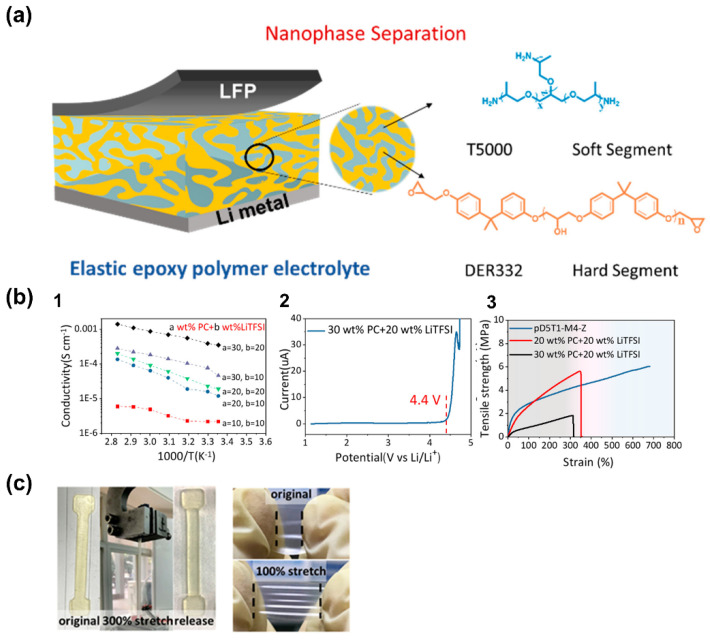
(**a**) Schematic representation of the developed GPE by nanophase-induced phase separation; (**b**) 1: ionic conductivities as a function of temperature for different contents of PC and LiTFSI salt; 2: cyclic voltammetry for the optimized GPE with 30 wt.% PC + 20 wt.% LiTFSI; 3: stress–strain curves; (**c**) photographs of tensile tests. Reprinted with permission from [90]. Copyright 2021 American Chemical Society.

Another technique, namely, polymerization with induced phase separation, has been applied to the development of versatile polymer electrolytes by creating two percolating phases during polymerization in the presence of a liquid phase, typically composed of ionic liquids with dissolved Li^+^ salts. The resultant material is composed of a mechanically robust phase and an ion conductive phase, ensuring both stiffness and conductivity for improved battery performance safety and stability [109,110,111]. Nanophase-separated epoxy polymers were recently developed by Zeng et al. [90] by combining an elastic resin composed of hard and soft segments that polymerizes into two diverse phases after curing, as represented in Figure 12a. The latter is responsible for creating an ionic pathway and the former produces mechanical integrity.

After swelling in different ratios of propylene carbonate (PC) solution mixed with LiTFSI salt, the resulting GPE containing an optimal 30 wt.% PC + 20 wt.% LiTFSI delivered an ionic conductivity of 3.5 × 10^−4^ S·cm^−1^ at RT, as well as wide electrochemical stability of up to 4.4 V (Figure 12(b1,b2)). Despite gradually reducing the membrane’s mechanical properties, an acceptable stress of 1.89 MPa and a toughness of 3.4 MJ·m^−3^ was also achieved with the addition of a plasticizer (Figure 12(b3)). These opposite characteristics were achieved with a bisphenol A diglycidyl ether (DGEBA) robust matrix in combination with flexible poly(propylene glycol) (PPG) moieties. This study demonstrated that phase separation can be used to provide good ionic conductivity while ensuring mechanical integrity (Figure 12c). It is worth mentioning that the combination of these properties in a single membrane is currently the biggest impediment to the development of these types of materials.

#### 4.1.4. SIC (Single-Ion Conducting)

To develop new types of polymeric electrolytes exhibiting high conductivity and good mechanical properties, dendrite suppression is a fundamental aspect to consider during the material design to ensure good safety of the overall system. According to the current state of the art, SPEs show dual ionic conduction, as lithium ions and their respective counter ions migrate during charge and discharge cycles between the cathode and anode, displaying lower t_Li_+. In these materials, only 20% of the ionic conductivity is due to the Li mobility, resulting in an enhancement of the ionic concentration gradient and, consequently, promoting an increase in cell polarization during the discharge process. This effect limits the amount of current available and decreases the lifetime of the battery [112].

**Figure 13:**
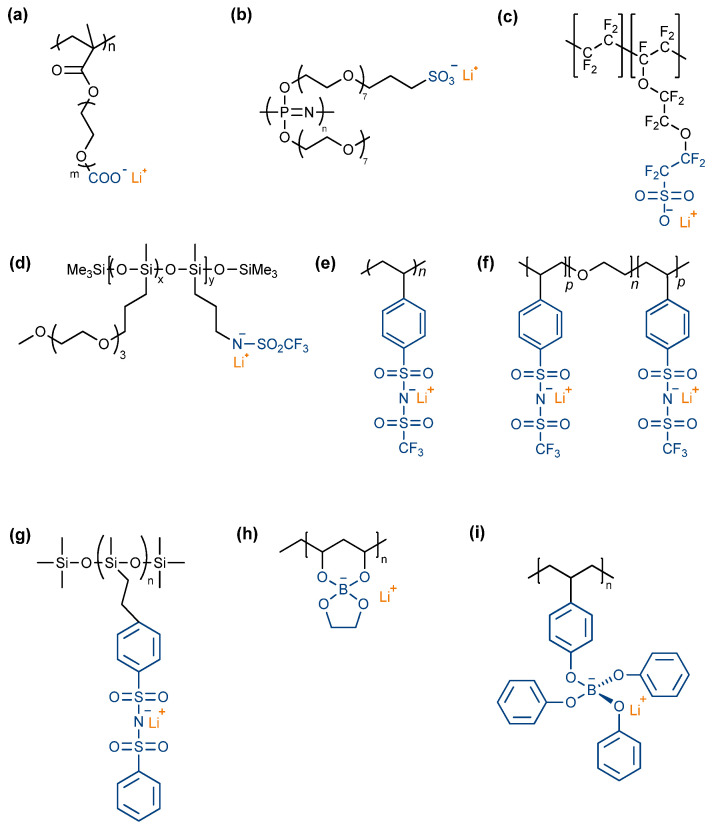
Chemical structures of different single-ion polymer electrolytes. SICs are typically constituted by strong electron-withdrawing groups ((**a**) [114]) that promote delocalization of negative charges, resulting in an increased dissociation and mobility of the Li salt. The most common examples are sulfonate (-SO3) ((**b**) [115], (**c**) [116,117]), sulfonylimide (-N(SO_2_)_2_-) ((**d**) [118], (**e**) [119], (**f**) [97], (**g**) [120]), and tetrahedral borate (-BO_4_) ((**h**) [121], (**i**) [122]) anions.

This main drawback in traditional SPEs can be overcome by blocking the migration of Li counter ions, typically carried out by tethering the counter ions’ moieties to the polymer backbone, promoting the free migration of Li^+^ ions. The use of single-ion polymer electrolytes (SIC), which have a t_Li_+ close to the unity, has proved to be an efficient method for suppressing dendrites and increasing the battery’s life and safety, as the anion immobilization allows a free migration of Li^+^ ions and consequent decrease in cell polarization. Owing to the potential of SIC, a variety of reviews reported state-of-the-art developments over the last years focusing on this thematic [46,78,113]. Some interesting examples are shown in Figure 13, and the electrochemical behavior of SIC based on different ions is summarized in Table 4.

Several polymers containing sulfonate have been developed due to their easy preparation and acceptable delocalized structure, and thus provide reasonable electrochemical properties and improve safety. Yubin et al. [63] reported a diblock polymer based on a sulfonate anion that showed an RT conductivity of 1.95 × 10^−6^ S·cm^−1^ and a high t_Li+_ of 0.83, characteristic of a SIC polymer. The low RT conductivity of the copolymer was mostly due to the high association of Li ions to the tethered anion moieties, which resulted in poor ion solvation. To overcome this challenge, the authors proposed the addition of a plasticizer.

In recent studies, self-healing and stretchable polymer electrolytes exhibiting increased flame retardancy properties were developed by free radical polymerization of 2,2,3,4,4,4-hexafluorobutyl methacrylate (HFBM) and sulfobetaine (SBMA) monomers with sulfonate-containing groups [124]. Different self-healing SPEs were prepared by solution casting method using p(HFBM-co-SBMA)s with different SBMA mol%, (poly(HFBM)), EMI–TFSI, and LiTFSI salt with different weight ratios. Results show that at a higher mol% of sulfonate anions (above 3 mol%), the resulting conductivity dropped, which was associated with the strong ionic interactions of the anion with the Li cation. The electrolyte containing a 3 mol% ratio showed an ionic conductivity of around 10^−5^ S·cm^−1^; good mechanical performance, namely, tensile strength (>130 kPa) and stretching (>4000%); and enhanced flame retardancy. The results also evidence that a decrease in the *T_g_* occurred with the addition of ionic liquid owing to the lower crystalline regions, resulting in more Li^+^ migration and increasing the conductivity.

The assembled Li/LiFePO_4_ cell displayed a high discharge capacity of 144.8 mAh·g^−1^ (0.2 C rate), with 82% stability over 100 cycles. The self-healing ability was attributed to the imidazole moieties present in the EMI-TFSI ionic liquid, which formed ion–dipole interactions with the fluorine atoms of the polymer side chain, resulting in an impressive total repair and full recovery of its electrochemical and mechanical properties in less than 60 min.

**Figure 14:**
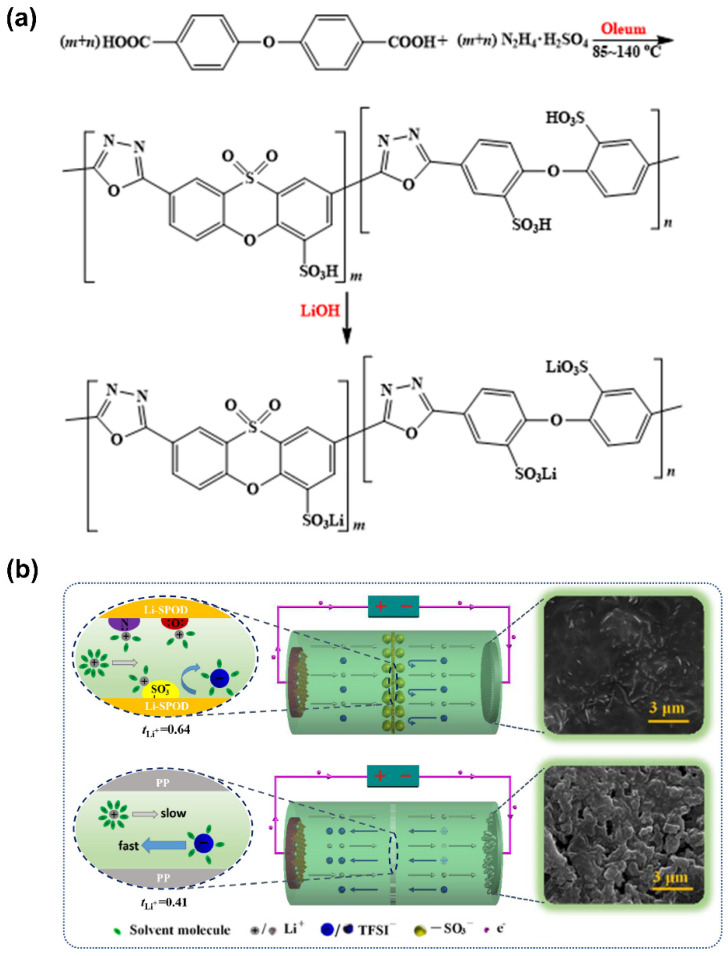
(**a**) Synthetic route of sulfonated aromatic polyoxadiazole (SPOD) polymer; (**b**) schematic representation of the ionic transport mechanism during charging for the studied Li/LiFePO_4_ cells with different SPOD or PP membrane and corresponding SEM images of the surface of Li anodes after 300 cycles at 2 C. Reprinted with permission from Li et al. [125]. Copyright 2021 Elsevier.

In addition, Li et al. [125] were able to develop a sulfonate-based polymer with oxadiazole moieties for the improvement of charge delocalization on the sulfonate groups, resulting in higher ion solvation. Sulfonated aromatic polyoxadiazole (SPOD) polymer was synthesized through a simple one-pot-method-based dicarboxylic acid (DPEA) and hydrazine sulfonate (HS) in oleum, followed by an ionic exchange with lithium hydroxide (Figure 14a).

Subsequently, the desired GPE membrane was prepared by electrospinning, followed by swelling in a 1 M LiTFSI-based liquid electrolyte. The obtained findings show that the Li-SPOD membrane presented a high degree of porosity, thus being capable of a higher liquid uptake compared to the traditional polypropylene (PP) separator (81.2% and 40.7%, respectively). The synthesis of a highly delocalized single-ion conductive structure was confirmed by electrochemical measurements, where the Li-SPOD GPE exhibited a superior 2.03 × 10^−3^ S·cm^−1^ ionic conductivity at RT, as well as a high t_Li_+ of 0.64 and good anodic compatibility.

Moreover, the freestanding electrospun membrane displayed a decent tensile strength of 14.2 MPa and good thermal stability up to 472 °C. The Li/LiFePO_4_ cell delivered an initial discharge capacity of 125 mAh·g^−1^ (2 C rate) and was capable of significantly suppressing the formation of dendrites on the metallic Li anode after 300 cycles, in comparison with the cell assembled with a PP separator that did not deliver the same suppression capacity, as can be seen in Figure 14b.

**Figure 15:**
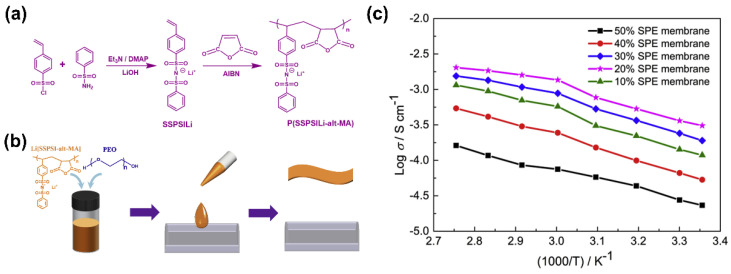
(**a**) Synthetic route of P(SSPSILi-alt-MA) polymer; (**b**) schematic representation of the electrolyte preparation; (**c**) ionic conductivities for the developed SPE with different SSPSILi contents. Reprinted with permission from Cao et al. [126]. Copyright 2021 Elsevier.

On the other hand, the sulfonylimide-based polymers have gained scientific attention due to their highly delocalized structure and weak ion pairing, which enables Li cation migration. Several studies have shown that this type of polymer, showing a good ionic conductivity and higher t_Li_+, has been achieved, making this type of material promising for future applications. Bis(benzene sulfonyl)imide was selected in several studies for the development of different SPEs, promoted by the higher delocalization of the bis(sulfonylimide) bond covalently linked to a benzene ring at both ends, with the effect of this polyanion on the conductivity and t_Li_+ of the SPE being notorious. Recently, in 2019, Cao et al. [126] showed a novel type of solid-state electrolyte based on lithium 4-styrenesulfonyl(phenylsulfonyl)imide (SSPSILi) and maleic anhydride (MA) (Figure 15a) [130]. The resulting alternating copolymer that employed both delocalized structures, attributed to the SSPSILi monomers, and ion solvating moieties (MA groups) was blended with PEO (Figure 15b) in different weight ratios to provide a decent ionic conductivity of 3.08 × 10^−4^ S·cm^−1^ at RT and high t_Li_+ of 0.97, without the incorporation of plasticizers (Figure 15c).

Blending with PEO provided a decrease in the crystallinity regions of the polymer, allowing the development of a homogeneous membrane without porosity and with increased ionic conductivity. The optimal SPE membrane containing 20 wt.% of the lithiated copolymer polymer displayed a higher electrochemical performance superior to the results attained so far using sulfonyl (trifluoromethyl-sulfonyl)imide-based SPEs, as well as dendrite suppression capacity due to the superior t_Li+_ and thermal stability up to 400 °C. These improved characteristics resulted in a corresponding Li/LiFePO_4_ cell with a charge–discharge capacity of 152.3 mAh·g^−1^ at 0.02 C, with 97.5% retaining capacity after 100 cycles. Superior stability at 0.1 C was achieved for over 300 cycles, which proves the improved cycling stability of the assembled cell.

**Figure 16:**
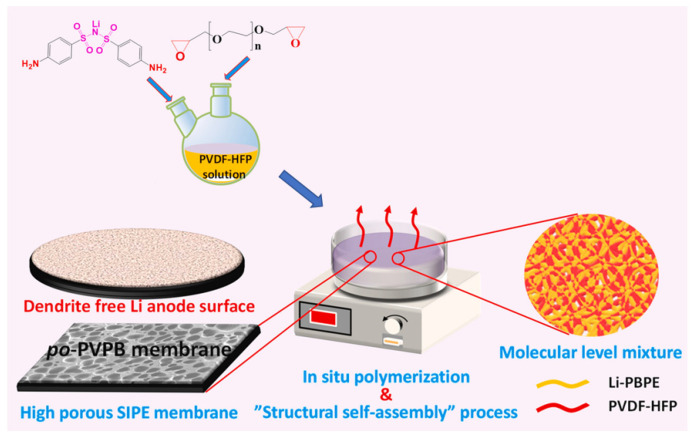
Schematic representation of the preparation of the SIPE membrane via a structural self-assembly process and in-situ polymerization. Reprinted with permission from Pan et al. [127]. Copyright 2021 Elsevier.

Additionally, the development of a SIC resin was recently developed by Pan et al. [127], based on bis(4-amino benzene sulfonyl)imide (Li-BABSI) and poly(ethylene glycol) diglycidyl ether (PEGDGE) monomers. The use of a rigid aromatic precursor containing delocalized -N(SO_2_)_2_ groups conferred superior mechanical performance to the membrane and high Li migration and t_Li_+, whereas the aliphatic monomers containing ether groups were responsible for an efficient Li^+^ conducting path. The developed polymer electrolyte was obtained by a “structural self-assembly” and in-situ polymerization process by first blending the polymerizable monomers with PVDF-HFP dissolved in N-methyl-2-pyrrolidone (NMP) solution, with subsequent polymerization at 100 °C (Figure 16).

After soaking with plasticizer, the resultant porous and flexible-yet-stiff membrane delivered a tensile strength of 10.5 MPa and high ionic conductivity of 2.3 × 10^−4^ S·cm^−1^ (at 25 °C). Furthermore, a t_Li_+ of 0.9 was obtained, even after 800 h, which was crucial to suppressing dendrite formation, which was further confirmed by the Li stripping/plating cycle test performed using an SPE-based assembled cell. An impressive discharge capacity of 165 mAh·g^−1^ at 0.05C was also obtained, which slightly differs from the cathode’s theoretical specific capacity value of 170 mAh·g^−1^. Good cycling stability after 200 cycles was also achieved, with 95.5% retention, as well as nearly 100% coulombic efficiency. This promising work provides a simplified process suitable for future structural industrial applications.

Another approach, based on this technique, used a Li (4-styrenesulfonyl) (trifluoromethanesulfonyl)imide monomer to develop highly crosslinked matrices with a superior electrochemical capacity [128]. UV-induced thiol-ene chemistry was implemented to efficiently synthesize the desired conductive polymer after soaking in plasticizer, resulting in a gel membrane that displayed an acceptable tensile strength of 2.8 MPa and superior thermal stability of 240 °C. This GPE also exhibited a high ionic conductivity of 8.4 × 10^−4^ S·cm^−1^ at 25 °C and superior 0.93 t_Li_+, which delivered an optimal initial discharge capacity of 133 mAh·g^−1^ (1.0 C rate) for the respective Li/LiFePO_4_ cell.

Moreover, apart from good rate capacity, the cell also exhibited superior cycling performance by keeping 83% of its retaining capacity after 400 cycles, with almost 100% coulombic efficiency.

Borate-based polymers have also been explored as potential electrolytes, due to boron’s low interaction with Li^+^ [78,131,132]. In a recent study by Zhang et al. [129], a conductive polymer was synthesized based on an aromatic *sp^3^* boron moiety (-B(O-)_4_). The developed poly (4,4′-dihydroxydiphenyl sulfone borate) polymer (Li-PSB) was mixed with polybenzimidazole (PBI) binder, constituted by imidazole and aromatic groups, which enabled Li solvation and rigidity of the membrane, respectively. The fabricated membranes were subsequently immersed in EC/DEC electrolyte to produce a GPE, displaying enhanced fire-retardancy properties, good ionic conductivity at RT, and a high transference number (up to 6.2 × 10^−4^ and t_Li_+ = 0.85, respectively). The incorporation of rigid aromaticity derived from the PBI binder resulted in a superior mechanical strength of 21.1 MPa. The electrochemical performance of the prepared Li/LFP resulting cell was also evaluated and a specific capacity of 131.8 mAh·g^−1^ (0.1 C rate) was found, as well as a 76.1% retaining capacity after 200 cycles, with nearly 100% coulombic efficiency. In this work, the inventors compared the developed GPE to a commercial separator and showed that their membrane presents superior benefits for fire retardancy, dendrite suppression, and higher ionic conductivity.

## 5. Polymer Composite Electrolytes

When the design of polymer electrolytes alone is not enough to achieve the desired ionic conductivity, different strategies can be approached, including the incorporation of inorganic fillers into the SPE matrix. These fillers are normally divided into two categories: active fillers and inactive (also known as inert or passive) fillers. The former are capable of conducting ions such Li^+^ themselves, and the latter, despite being ionic insulators, offer a favorable environment for ion transport in a polymer matrix. The incorporation of inactive fillers enhances the free Li^+^ mobility and suppresses the polymer crystallization.

The first study focused on the incorporation of inorganic (inactive) filler into a polymer electrolyte was in 1982, by Weston et al. [133]. The authors reinforced Li perchlorate–PEO polymer electrolyte with alpha-alumina (α-Al_2_O_3_), achieving a significant enhancement of the mechanical stability of the composite. However, ionic conductivity improvements by adding Al_2_O_3_ particles into a PEO-based matrix were first proposed by Wieczorek et al. [22] in 1989. Since then, the incorporation of a wide range of inorganic inactive fillers into polymer electrolyte matrices have been extensively studied [134,135,136,137,138].

For example, Sasikumar et al. [139] reported the fabrication of a flexible hybrid polymer electrolyte based on a PVDF-HFP and poly(vinyl acetate) polymer blend reinforced with titanium dioxide (TiO_2_) nanoceramic filler. The incorporation of TiO_2_ resulted in an improvement of the ionic conductivity (2.69 × 10^−3^ S·cm^−1^, at 30 °C), thermal stability (up to 350 °C), and mechanical strength (8.4 MPa). The TiO_2_ SPE also exhibited large t_Li_^+^ (0.53) as well as an extended electrochemical window of stability (5.4 V vs. Li/Li^+^).

**Figure 17:**
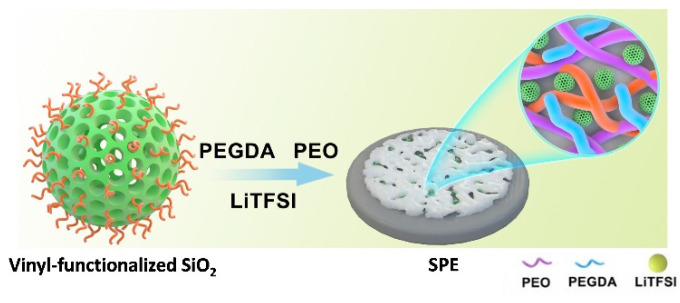
Schematic illustration of the vinyl-functionalized SiO_2_/PEO crosslinked SPE [140].

Moreover, Zhan et al. [140] prepared a partial crosslinked PEO-based SPE using porous vinyl-functionalized silicon dioxide (SiO_2_) as the filler and PEGDA as the crosslinker (Figure 17).

The combination of the mechanical rigidity of SiO_2_ fillers and the flexibility of the PEO promoted an enhancement of the mechanical properties and simultaneously the inhibition of the PEO recrystallization, promoting the dissolution of the Li^+^ salt. Furthermore, the SiO_2_-reinforced SPE membrane showed good ionic conductivity (5.08 × 10^−3^ S·cm^−1^ at 60 °C), a wider electrochemical window of stability (5.2 V vs. Li/Li^+^), and better ability to suppress dendrite growth.

Xu et al. [141] developed a flexible polypropylene oxide (PPO)-based SPE membrane by combining the bis[3 -(methyldimetoxysilyl)]-terminated PPO with ZrO_2_ fillers, succinonitrile as a plasticizer, and a cellulose membrane framework. The SPE membrane exhibited improved ionic conductivity (9.6 × 10^−4^ S·cm^−1^, at RT), large t_Li_+ (0.8), and a high potential window (5.0 V vs. Li/Li^+^).

In another recent study, a boron nitride (BN)-based SPE was developed [142]. The crosslinked polymer electrolyte was prepared using cellulose acetate as a matrix and PEGDA as a crosslinking agent. The resulting SPE membrane showed high ionic conductivity (8.9 × 10^−3^ S·cm^−1^ at 30 °C), excellent electrochemical stability up to 5.5 V vs. Li/Li+, and good thermal stability. A wide range of other inorganic inactive fillers can be found in the literature as potential reinforcements for SPE membranes, including cerium dioxide (CeO_2_) [82], molybdenum disulfide (MoS_2_) [143], silicon nitride (Si_3_N_4_) [144], niobium pentoxide (Nb_2_O_5_) [145], graphitic carbon nitride (g-C_3_N_4_) [146], quantum dots (QD) [147], nitrogen and sulfur co-doped carbon dots (NS-CD) [148], and aluminosilicate zeolite (SSZ-13) [149], among others.

**Figure 18:**
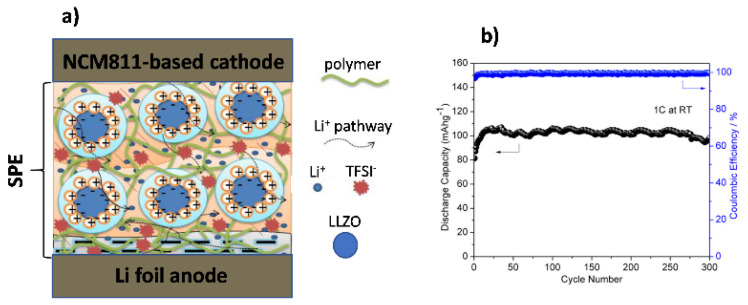
(**a**) Schematic illustration of the structure of the cell; (**b**) cycling performance of the full cell at 1C, room temperature. Reproduced with permission from Walle et al. [152]. Copyright 2021 American Chemical Society.

Active fillers are more effective at boosting the electrochemical performance of SPEs since they can conduct Li^+^ ions. The most used are garnet-type (aluminum-doped lithium lanthanum zirconate oxide (LLZO) and its derivatives, such as tantalum-doped lithium lanthanum zirconate oxide (LLZTO)), NASICON-type (lithium aluminum titanium phosphate (LATP), among others), and perovskite-type (such as lithium lanthanum titanate (LLTO)) [150,151,152,153]. Walle et al. [152,154] developed a co-precipitation method in a Taylor flow reactor to synthesize separately both the cathode, the nickel-rich hydroxide Ni_0.8_ Co_0.1_ Mn_0.1_ (OH)_2_ (NCN811), and the electrolyte, an LLZO-reinforced PVDF/PAN/LiTFSI polymer electrolyte matrix (Figure 18). The SPE membrane exhibited good ionic conductivity (4.50 × 10^−4^ S·cm^−1^), high t_Li_+ (0.84), and a broader electrochemical window (5.04 V vs. Li/Li^+^). A CR2032 coin cell containing Li/NCM811 achieved a capacity retention of 89.8% after 300 cycles at 1C and RT. Figure 18a shows an illustration of the cell structure and Figure 18b represents the cycling results of the full cell at 1C, RT.

Jin et al. [151] reported a structural design of SPE consisting of three-dimensional (3D) interconnected LATP, PVDF, and LiTFSI. The LATP filler was coated with polymethyl methacrylate (PMMA), which acted as a functional modification layer of LATP and improved the interface with the PVDF matrix. The SPE membrane demonstrated enhanced ionic conductivity (1.23 × 10^−3^ S·cm^−1^ at RT) and a remarkable t_Li_+ (0.85). Recently, a new type of Li-ion conductor oxide with trivalent gallium metal (LLGO) was also studied as an active filler for incorporation into SPEs. [155] Using LLGO as a conducting filler, the PEO/LiTFSI/LLGO SPE composite exhibited good ionic conductivity (4.4 × 10^−4^ S·cm^−1^ at 60 °C) and a t_Li_+ as high as 0.69.

Although active fillers possess an ionic conductivity in the range of 10^−4^–10^−3^ S·cm^−1^, a wide electrochemical window, and enhanced thermal stability, they also show some drawbacks. For example, NASICON-type and perovskite-type reinforced electrolytes can be easily reduced by Li metal, and garnet-type reinforced electrolytes are unstable under air and easily react with water and carbon dioxide, generating lithium carbonate (Li_2_CO_3_) and lithium hydroxide (LiOH) [124].

**Figure 19:**
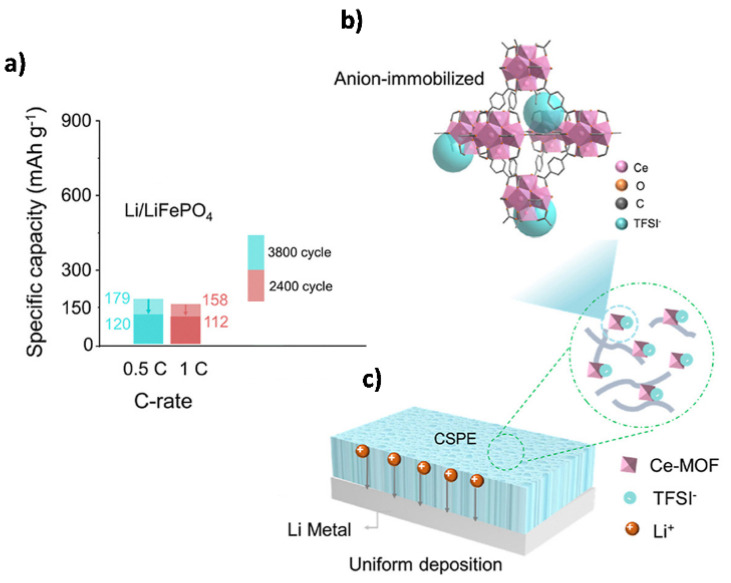
(**a**) Specific capacity of the Li/Ce-MOF SPE/FFP cell; schematic illustration of (**b**) anion immobilization on Ce-MOF fillers and (**c**) evolution of Li plating modulated by Ce-MOF SPE during cycling. Reproduced with permission from Wu et al. [158]. Copyright 2021 Elsevier.

Recently, metal–organic frameworks (MOF) consisting of metal nodes and organic ligands have attracted significant interest as fillers in SPEs. The high specific surface area of MOFs combined with their highly ordered crystal structures and porosity make these materials suitable for adsorbing liquid electrolytes and regulating ion flux. Furthermore, the organic–inorganic hybrid nature of MOFs can improve compatibility with SPEs [156]. Zhang et al. [157] developed an MOF-derived cobalt-doped hollow porous carbon nanocage (Co-MOF), which was capable of absorbing Li^+^-containing ionic liquid. The Co-MOF-reinforced PEO/LiTFSI SPE membrane showed high ionic conductivity (1.91 × 10^−4^ S·cm^−1^, at 30 °C), wide electrochemical stability (5.2 V vs. Li/Li^+^), and high t_Li_+ (0.5). In another attempt, Wu et al. [158] incorporated a 3D-structured cerium-based MOF (Ce-MOF) nanofiller into a PEO/LiTFSI polymer electrolyte. The abundant cavities of the Ce-MOF enabled the strong Lewis acid–base interactions with both oxygen in the PEO chain and the anion in the Li salt, leading to an improved ionic conductivity (3.0 × 10^−5^ S·cm^−1^ at 30 °C) and high t_Li_+ (0.75). The Ce-MOF SPE exhibited superior cycling stability, with a capacity of 120 mAh·g^−1^ after 3800 cycles when tested at 0.5 C, 60 °C, for a Li/LiFePO_4_ cell. Figure 19a presents the specific capacity of the Li/Ce-MOF SPE/FFP cell, and Figure 19b,c illustrates a scheme of the anion immobilization and the evolution of Li plating during cycling.

In addition, Zhang et al. [159] prepared a copper-based MOF (Cu-MOF)-supported PEO polymer electrolyte by UVcuring, using benzophenone as a photoinitiator. The Cu-MOF SPE showed high ionic conductivity (4.99 × 10^−3^ S·cm^−1^, at 30 °C), a wide electrochemical window (5.25 V vs. Li/Li^+^), and a high t_Li_+ (0.61). It also exhibited good cycle stability in symmetric battery, with 98.6% capacity retention after 700 cycles (tested at 1C at RT).

Table 5 summarizes the performance parameters of the latest studies performed on some inorganic filler-reinforced polymer-based electrolytes.

## 6. Application of Polymer Electrolytes for Structural Batteries

**Figure 20:**
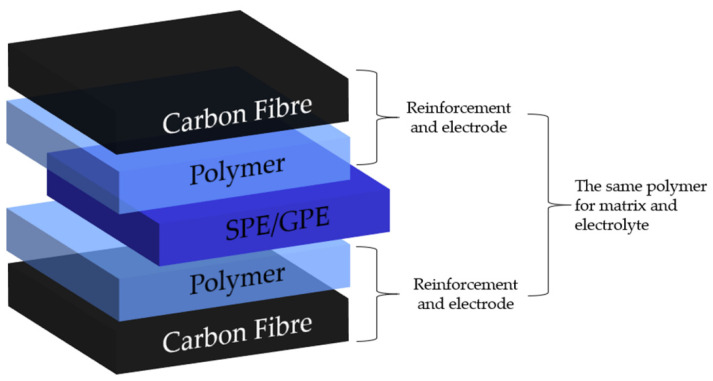
Representation of a structural battery concept.

Since the application of a liquid electrolyte is detrimental from a mechanical point of view, one of the main applications of SPEs/GPEs is their employment in structural batteries. These multifunctional devices are classified as a class of mass-less structural composites with high-density energy storage systems with enhanced mechanical properties [148,174]. In the development of a structural battery composite material, the challenging factor relies on making every constituent play multiple functions [3]. This means, for example, that carbon fibers (CFs) work as both electrodes and structural reinforcements, and polymer can act as matrix and electrolyte simultaneously. CFs are promising materials that have been studied as multifunctional electrodes. This is possible due to the enhanced mechanical, electrical, and electrochemical properties of CFs. Figure 20 shows a representative scheme of a structural battery.

A structural battery can be produced using two main approaches: (i) through the incorporation of the functionalities into a component by, for example, making a laminated composite structure, and (ii) through the transformation of each component in a multifunctional material. In the first case, the electrolyte is capable of mechanical load transfer and is called a structural battery electrolyte (SBE). In the second case, the approach relies on an assembly of different constituents rather than a device with multifunctional components [174].

CFs are used as anodes due to their high tensile strength (2–4 GPa), high electrical conductivity (4.9 S·cm^−1^), and great Li^+^ insertion capacity (280 mAh·g^−1^) [63,175,176,177,178]. For structural positive electrodes, CFs can also be employed as a cathode material. The very first effort in the development of a laminated structural composite takes us back to 2007, to the U.S Army Research Laboratory, where a metal mesh with cathode material was employed to be used as positive electrode and CFs as the negative electrode, both separated by a glass fiber (GF) weave and a common polymer electrolyte matrix [3,148,179]. Although the resulting device showed good mechanical properties, poor electrical insulation led to the incapability of electrochemical storage. This pioneering work boosted many efforts in this field. In 2009, a structural battery with short-fiber reinforced electrodes and an SPE matrix was designed by Liu et al. [180]. However, the authors did not find a sufficiently good SPE to conduct ions and used a GPE instead. Poor mechanical properties were also achieved since the employed GPE had a low tensile modulus (3 MPa), and the resulting battery showed an energy density of 35 Wh·kg^−1^. In 2010, Eksted et al. [181] developed another attempt through the employment of a GPE reinforced with a CF weave anode and a LiFePO_4_ fiber weave as a cathode. Unfortunately, the mechanical properties attained were unacceptable and no electrochemical data were reported.

Therefore, the challenge relies on the development of a safe solid/gel electrolyte able to efficiently conduct ions (up to ~1 mS·cm^−1^) and withstand mechanical load (shear stiffness between 0.1 and 1 GPa) [1,182]. Another important parameter to achieve is a high Young modulus, which means that if the electrolyte has a low modulus it will not be able to load mechanical transfer between fibers, which is a crucial attribute of the matrix in a structural composite [148]. Each component, from electrodes and electrolytes to separators, can be optimized to provide higher strength to the final battery and the overall stiffness should minimally decrease the power and energy density to justify the multifunctionality [63].

Table 6 summarizes some composite solid-state electrolytes with a wide electrochemical window of stability employed to develop structural batteries. An overview of the literature revealed that only a few reports are available on batteries with high-voltage cathodes (NMC811 and NMC622, for example) [183].

Other interesting examples reported the multifunctional mechanical properties of structural batteries. For instance, in 2009, Liu et al. [180] established a structural battery with carbon nanofibers with tunable mechanical properties. The electrolyte was PVDF-based, along with a graphitic anode and lithium cobalt oxide (LiCoO_2_) as the cathode. The final assessment showed good mechanical properties with a 3.1 GPa tensile modulus but low specific energy (35 Wh·kg^−1^, at a 0.05C discharge rate) [148,192,193]. Another tactic was underlined by Ihrner et al. [192] and subsequently optimized by Schneider et al. [63,194]. A porous methacrylate polymer impregnated with a liquid mixture containing Li^+^ salts was used as an electrolyte with an ionic conductivity of 2 × 10^−4^ S·cm^−1^ and a Young’s modulus of 0.5 GPa [148,193]. Moyer et al. [194] successfully produced a structural pouch cell with an energy density of 25 Wh·kg^−1^ at 0.1 C and mechanical strength of 213 MPa. In this case, a graphitic anode and a CF coated LFP cathode were used to serve as current collectors.

There is still ample room available for optimizations in this field, especially concerning the mechanical point of view of a structural battery as well as the necessary characterizations.

## 7. Summary

In recent years, significant research and progress in the field of solid-polymer electrolytes have been made. In this review, the recent advancements and research performed on the design of solid- and gel-polymer electrolytes were summarized, as well as composite polymer electrolytes, focusing their application on LIBs. Compared to liquid electrolytes, SPEs have the potential to offer more balanced overall battery performance in terms of their nonflammability, mechanical properties, ionic conductivity, safety, and thermal stability. The current limitations to SPE design were also outlined. Although solid-polymer electrolytes have shown great potential, challenges still need to be addressed to fulfil the optimized performance for practical applications. Strategies such as polymer blending, copolymerization, polymer crosslinking, and the development of single-ion polymer electrolytes, consisting of tethering the Li counter ion moieties to the polymer backbone, have been explored, aiming at optimizing SPE. Some key factors to be considered when designing SPE include (i) enhanced ionic conductivity at room temperature—it is still fairly challenging to achieve solvent-free polymer electrolytes with an ionic conductivity higher than 10^−3^ S·cm^−1^; (ii) improved t_Li+_, as it favors the reduction of polarization and suppression Li dendrite growth; (iii) optimized interfaces between electrodes and electrolytes and an understanding of their interfacial behavior; (iv) enhanced electrochemical and thermal stabilities of SPEs to boost their lifespan; (v) low-cost preparation techniques for SPE as well as the sustainability of their polymer batteries for commercial applications; and (vi) mechanical stability for polymer electrolytes. The electrolyte must not be brittle, should be flexible and elastic, and should also stand the stress conditions during their lifespan. Despite the challenges outlined above, SPEs have been broadly recognized as one of the core directions to enhance the performance and safety of LIB. Bearing in mind the intense research and industrial interest in developing SPE and solid-state batteries, it is entirely reasonable to expect significant advances in the field to continue to occur.

## Figures and Tables

**Table 1:** Overview of inherent properties of the employed electrolytes in LIBs.

Electrolyte Type	Ionic Conductivity (RT)	Interfacial Properties	Thermal Stability	Electrochemical Stability	Mechanical Strength	Safety
Liquid	>10^−3^ S·cm^−1^	Good	Poor	Poor	Poor	Poor
Solid polymer	<10^−4^ S·cm^−1^	Poor	Good	Good	Good	Good
Gel polymer	>10^−4^ S·cm^−1^	Medium	Medium	Poor	Medium	Medium

**Table 2:** PEO/lithium perchlorate (LiClO_4_), PEO-tetraethylene glycol dimethacrylate (TEGDMA)—tetraethylene glycol dimethyl ether (TEDME)/lithium bis(trifluoromethanesulfonyl)imide (LiTFSI), PEO-sulfur-poly(ethylene glycol) methacrylate) (PEGMA)/LiTFSI, and polyethylene glycol (PEG)-hexamethylene diisocyanate trimer (HDIt)/LiTFSI optimization strategies.

Polymer	Optimization Strategy	Ionic Conductivity (S·cm^−1^)	EWS (V vs. Li/Li^+^)	t_Li_+	Year	Ref.
PEO/LiClO_4_	PEO/LiX (X:ClO_4_^−^)	1.03 × 10^−5^, 30 °C	-	0.21	2005	[60]
(PEO-TEGDMA-TEGDME)/LiTFSI	Crosslinking	2.70 × 10^−4^, 24 °C	5	0.56	2019	[61]
(PEO-sulfur-PEGMA)/LiTFSI	Polymer blending	2.13 × 10^−4^, 50 °C	5.4	0.61	2020	[62]
(PEG-HDIt)/LiTFSI	Copolymerization	6.51 × 10^−5^, 25 °C	4.65	0.49	2021	[63]

**Table 3:** Reported electrochemical parameters of different polymer electrolytes designed by different approaches.

Structure	σ (S·cm^−1^) (°C)	t_Li_+	Cell Type	Initial Capacity (mAh·g^−1^) (Conditions)	Cycle Stability (Capacity Retention)	Ref.
PEO/PVA	1.25 × 10^−4^ (25 °C)	-	-	-	-	[79]
PVDF-HFP/PETEA	5 × 10^−4^ (25 °C)	-	Li/LiFePO_4_	151 (0.5 C, 25 °C)	50 (98%)	[80]
PVDF/TMS	3.21 × 10^−3^ (25 °C)	0.03	Li/LiFePO_4_	181 (0.1 C, 25 °C)	50 (91%)	[81]
PVDF/PAN-POSS	1.91 × 10^−3^ (20 °C)	-	Li/LiFePO_4_	112 (0.1 C, 20 °C)	25 (~90%)	[82]
P(HOEA-co-MA)-PEG	6.0 × 10^−4^ (25 °C)	0.35	Li/LiFePO_4_	101.4 (2.0 C, 22 °C)	1000 (66%)	[83]
P(PEGMA-co-MMA)	3.02 × 10^−5^ (30 °C)	0.37	Li/LiFePO_4_	166.5 (0.2 C, 60 °C)	290 (93%)	[84]
PCL-PPC-PCL	3 × 10^−5^ (30 °C)	0.4	Li/LiFePO_4_	142 (0.05 C, 30 °C)	200 (90%)	[85]
CN/FM-PAGE	1.01 × 10^−4^ (30 °C)	-	Li/S	944 (0.2 C, 60 °C)	80 (~60%)	[86]
PEGMEA-PEGDME-XVIm-TFSI	3.16 × 10^−4^ (25 °C)	-	Li/LiFePO_4_	160 (0.2 C, 25 °C)	150 (93.8%)	[87]
PEG-BTA	4.79 × 10^−3^ (30 °C)	0.38	Li/LiFePO_4_	118.2 (5 C, 30 °C)	125 (97.8%)	[88]
VEMI-TFSI-PEI-PEGDGA	1.03 × 10^−3^ (25 °C)	0.47	Li/LiFePO_4_	165.6 (0.1 C, 25 °C)	200 (97.3%)	[89]
T5000-DER332 (epoxy resin)	3.5 × 10^−4^ (25 °C)	-	Li/LiFePO_4_	130 (0.1 C, 25 °C)	120 (93%)	[90]

**Table 4:** Reported electrochemical parameters of polymeric single-ion electrolytes based on different anions.

Anion	σ (S·cm^−1^) (°C)	t_Li_+	Cell Type	Initial Capacity (mAh·g^−1^) (Conditions)	Cycle Stability (Capacity Retention)	Ref.
–SO_3_	1.95 × 10^−6^ (25 °C)	0.83	Li/LiFePO_4_	-	-	[123]
–SO_3_	1.2 × 10^−5^ (25 °C)	0.37	Li/LiFePO_4_	144.8 (0.2 C, 25 °C)	100 (82%)	[124]
–SO_3_	2.03 × 10^−3^ (25 °C)	0.64	Li/LiFePO_4_	125 (2.0 C, 25 °C)	300 (99%)	[125]
–N(SO_2_)_2_	3.08 × 10^−4^ (25 °C)	0.97	Li/LiFePO_4_	152.3 (0.02 C, 25 °C)	100 (97.5%)	[126]
–N(SO_2_)_2_	2.3 × 10^−4^ (25 °C)	0.9	Li/LiFePO_4_	165 (0.05 C, 25 °C)	200 (95.5%)	[127]
–N(SO_2_)_2_	8.4 × 10^−4^ (25 °C)	0.93	Li/LiFePO_4_	133 (1.0 C, 25 °C)	400 (83%)	[128]
–BO_4_	6.2 × 10^−4^ (25 °C)	0.85	Li/LiFePO_4_	131.8 (0.1 C, 25 °C)	200 (76.1%)	[129]

**Table 5:** Performance parameters of the latest studies performed on inorganic filler-reinforced polymer-based electrolytes.

Filler	SPE	σ (S·cm^−1^) (°C)	t_Li_+	EWS (V vs. Li/Li^+^)	Discharge Capacity (mAh·g^−1^) (Conditions)	Cycle Stability (Capacity Retention)	Tensile Strength (MPa)	Ref.
TiO_2_	PVDF/PVA/LiTFSI	2.69 × 10^−3^ (30 °C)	0.53	5.4	154 (0.1 C; 40 °C)	50 (94%)	8.4	[139]
TiO_2_	PVDF/LiTFSI	1.51 × 10^−3^ (30 °C)	0.42	-	160 (0.1 C; 60 °C)	50 (80%)	0.9	[76]
TiO_2_ Nanowires	PEO/LiTFSI	1.10 × 10^−4^ (30 °C)	0.36	5.5	151 (0.1 C; 60 °C)	100 (91%)	-	[65]
SiO_2_	PEO/LiTFSI	7.5 × 10^−6^ (25 °C) 4.3 × 10^−4^ (60 C)	-	-	150 (0.1 C; 60 °C)	80 (88.4%)	2.3	[154]
SiO_2_	PEO/PEGDA/LiTFSI	5.08 × 10^−3^ (60 °C)	-	5.2	155.1 (0.5 C; 60 °C)	300 (91%)	2.46	[140]
SiO_2_	PEO/PEGDA/LiTFSI	3.37 × 10^−4^ (25 °C) 1.73 × 10^−4^ (0 °C)	0.38	4.9	129.4 (0.2 C; 0 °C)	150 (99%)	26.9	[160]
SiO_2_	PVEC/LiTFSI	1.65 × 10^−4^ (25 °C)	0.63	5.3	150 (0.5 C; 25 °C)	200 (79.4%)	-	[161]
SiO_2_	PPC/LiTFSI	8.48 × 10^−4^ (60 °C)	0.86	4.8	171 (0.1 C; 60 °C)	100 (86%)	4.0	[162]
SiO_2_	PEO/AKP/LiTFSI	2.52 × 10^−4^ (40 °C)	0.22	5.1	154 (0.5 C; 50 °C)	100 (97.9%)	5.51	[163]
SiO_2_@BN	PEO/LiTFSI	4.53 × 10^−4^ (60 °C)	0.54	4.71	150 (1.0 C; 60 °C)	900 (87%)	1.33	[164]
Si Nanotubes	PEO/LiTFSI	4.35 × 10^−4^ (30 °C)	0.65	5.0	151 (0.1 C; 60 °C)	100 (83.4%)	-	[25]
Al_2_O_3_	PVDF/LiPF_6_	8.5 × 10^−4^ (25 °C)	0.92	5.2	116 (2.0C; 25 °C)	2000 (88%)	27.1	[66]
ZrO_2_	PPO LiTFSI	9.62 × 10^−4^ (25 °C)	0.8	5.0	145 (0.5 C; 25 °C)	140 (63%)	47.5	[141]
ZrO_2_	P(S-MMA)/PVDF/LiClO_4_	1.2 × 10^−2^ (30 °C)	-	4.6	144 (0.1 C; 25 °C)	50 (74.3%)	-	[165]
CeO_2_	PEO/LiTFSI	1.1 × 10^−3^ (60 °C)	0.47	5.1	164 (0.1 C; 60 °C)	100 (98%)	0.6	[82]
BN	PEGDA/CA/LiPF_6_	8.8 × 10^−3^ (30 °C)	-	5.5	113.2 (0.1 C; 25 °C)	200 (77%)	-	[142]
MoS_2_	PVDF/LiTFSI	2.8 × 10^−4^ (25 °C)		4.57	137 (0.54 C; 25 °C)	200 (98%)	-	[143]
SSZ-13	PEO/LiTFSI	6.16 × 10^−4^ (30 °C) 5.34 × 10^−2^ (70 °C)	0.85	4.3	154 (0.1 C; 60 °C)	80 (94%)	5.3	[149]
Si_3_N_4_	PVDF/LiPF_6_	8.84 × 10^−4^ (25 °C)	-	4.25	146.3 (0.5 C; 25 °C)	100 (97.6%)	3.13	[144]
Nb_2_O_5_	PVDF/LiTFSI	6.6 × 10^−5^ (25 °C)	-	5.1	151 (0.5 C; 30 °C)	230 (~97%)	~9.2	[145]
g-C_3_N_4_	PEO/LiTFSI	3.18 × 10^−5^ (25 °C) 2.5 × 10^−4^ (60 °C)	0.69	5.2	168 (0.3 C; 60 °C)	400 (67%)	3.97	[146]
NS-CD	PEO/LiClO_4_	2.10 × 10^−4^ (25 °C)	0.51	5.0	145.2 (0.5 C; 45 °C)	200 (~95%)	2.51	[148]
QD	PEO/PLSS	2.02 × 10^−4^ (25 °C)	0.94	4.4	155.9 (0.2 C; 60 °C) 136.7 (2 C; 60 °C)	100 (94.3%) 1000 (83.4%)	5.1	[147]
LLZO	PVDF/LiClO_4_	1.2 × 10^−4^ (25 °C)	0.42	4.2	160.92 (0.1 C; 25 °C)	100 (92.5%)	-	[166]
LLZO	PVDF/PAN/LiTFSI	4.5 × 10^−4^ (25 °C)	0.84	5.0	103.8 (1.0 C; 25 °C)	300 (89.8%)	-	[152]
LLZO	PVDF/LiTFSI	1.74 × 10^−4^ (25 °C)	0.34	4.5	151 (0.5 C; 25 °C)	100 (71.5%)	95	[167]
LLZTO	PAN/PEO/LiTFSI	2.6 × 10^−4^ (30 °C)	-	4.5	170.1 (0.1 C; 30 °C)	100 (72.8%)	-	[168]
LLZTO	PEGDA/LiTFSI	3.1 × 10^−4^ (25 °C)	0.43	4.7	175 (0.2 C; 25 °C)	200 (85.4%)	-	[169]
LLZTO	PEO/LiTFSI	4.61 × 10^−4^ (60 °C)	-	5.1	130.3 (0.5 C; 60 °C)	200 (98.8%)	-	[170]
LLZTO	PPC/LiTFSI	2.7 × 10^−4^ (30 °C)	0.54	4.4	163 (0.2 C; 25 °C)	140 (90.79%)	11.78	[171]
LLZTO	PEO/LiTFSI	1.43 × 10^−3^ (25 °C)	-	4.8	152 (1.0 C; 25 °C)	1000 (90%)	0.55	[142]
LLZTO	PEO/LiTFSI	1.76 × 10^−4^ (30 °C)	0.53	5.2	155.3 (0.2 C; 60 °C) 136.1 (0.5 C; 60 °C) 120.7 (1.0 C; 60 °C)	300 (93.2%) 400 (90.6%) 1000 (86.0%)	9.47	[153]
LLTO	PAN/LiClO_4_	3.6 × 10^−4^ (25 °C)	0.38	5.0	142.5 (0.5 C; 25 °C)	100 (90%)	5.61	[150]
LLGO	PEO/LiTFSI	4.4 × 10^−4^ (60° C)	0.69	4.8	120 (0.2 C; 60 °C)	400 (80%)	1.3	[155]
LATP	PVDF/LiTFSI	1.23 × 10^−3^ (25 °C)	0.85	4.8	131.8 (0.5 C; 25 °C)	150 (91.2%)	5.68	[151]
LATP-GF	PEO/LiTFSI	6.3 × 10^−5^ (25 °C)	0.37	4.4	148 (0.5 C; 60 °C)	270 (68%)	33.1	[143]
LATP:LLTO	PVDF/LiClO_4_	2.08 × 10^−3^ (25 °C)	0.88	5.3	159.7 (0.1 C; 30 °C)	1000 (85%)	4.5	[172]
Co-MOF	PEO/LiTFSI	1.91 × 10^−4^ (30 °C)	0.5	5.2	140 (0.5 C; 60 °C)	150 (95.9%)	3.72	[157]
Ce-MOF	PEO/LiTFSI	2.76 × 10^−4^ (60 °C)	0.47	4.8	161.3 (0.5 C; 60 °C)	2000 (63%)	1.0	[173]
Ce-MOF	PEO/LiTFSI	3.0 × 10^−5^ (30 °C)	0.75	4.5	179 (0.5 C; 60 °C)	3800 (67%)	0.6	[158]
Cu-MOF	PEO/LiPF_6_	4.99 × 10^−3^ (30 °C)	0.61	5.25	144.6 (0.2 C; 25 °C) 138.3 (1.0 C; 25 °C)	200 (92.3%) 700 (98.6%)	-	[159]

**Table 6:** Summary of structural batteries, based on [184].

Electrolyte Type	Cathode Material and Mass Loading	σ (S·cm^−1^) (°C)	t_Li_+	EWS (V)	Discharge Capacity (mAh·g^−1^) (Conditions)	Coulombic Efficiency	Cycle Stability (Capacity Retention)	Ref.
Multilayer with PAN@LAGP-LiTFSI and PEGDA-LiTFSI	NMC811/NMC622/Super P/PVDF/PAN 3–5 mg·cm^−2^	3.7 × 10^−6^ (25 °C)	-	5	Li/NMC811 175 (25 °C, 0.5 C); Li/NMC622 180 (25 °C, 0.1 C);	-Li/NMC622 (25 °C, 0.5 C): 99.8%	Li/NMC811 81.5% Li/NMC622 97.7%	[185]
PVDF-5 wt% palygorskite ((Mg, Al)_2_Si_4_O_10_(OH)) nanowires-LiClO_4_	NMC111/Super C65/PVDF/LiClO_4_ 1.5 mg·cm^−2^	1.2 × 10^−4^ (25 °C)	0.54	4.7	117.6 (25 °C, 0.3 C, 3–4.2 V)	Close to 100%		[186]
PVDF-HFP-20% Li_6.4_Ga_0.2_La_3_Zr_2_O,- TEP and FEC (7:3, *v/v*)-LiFSI	NMC523/PVDF/Super-P 5 mg·cm^−2^	1.84 × 10^−3^ (20 °C)	0.563	4.75	96.3 (25 °C, 0.5 C, 2.8–4.3 V)	98%	200 (94.08%)	[187]
PVDF-HFP-12.5 wt% LLZTO-LiTFSI	LFP/PVDF-HFP/acetylene black 1.5 mg·cm^−2^	1.2 × 10^−4^ (30 °C)	0.33	5.6	60 °C, 0.5 C, 2.8–3.8 V	99%	50 (92.1%)	[188]
EGPEA-Nano fumed SiO_2_-LiTFSI	LFP/PVDF/KB 2.5 mg·cm^−2^	2.16 × 10^−5^ (25 °C)	0.63 (55 °C)	4.8	55 °C, 0.1 C, 2.5–4.0 V	-	100 (95%)	[189]
PEO -12.5 vol% UiO-66-LiTFSI	LFMP/LFP/LiTFSI/PEO/Super-P 2 mg·cm^−2^	3.1 × 10^−5^ (25 °C)	0.72	4.97	Li/LFP (60 °C, 1 C, 2.8–3.8 V): Li/LFMP (60 °C, 1 C, 2.8–4.4 V)	-	Li/LFP300 (85.4%) Li/LFMP 100 (81.2%)	[190]
PEO-20% P(SSPSILi-alt-MA)	LFP/P(SSPSILi-alt-MA)/PEO/ carbon black	3.08 × 10^−4^ (25 °C)	0.97 (at 80°C)	5.0	80 °C, 0.1 C, 2.5–4 V	~100%	100 (97.5%)	[126]
PEO-g-C_3_N_4_-LiTFSI	LFP/PVDF/carbon black 1.2 mg·cm^−2^	1.7 × 10^−5^ (30 °C)	0.56	4.7	60 °C, 0.2 C, 2.8–4.0 V	99.5%	100 (96.2%)	[191]

Note: LAGP—lithium aluminum germanium phosphate; Li_6.4_Ga_0.2_La_3_Zr_2_O—lithium lanthanum zirconium oxide doped with gallium (LLZO-Ga); TEP—triethyl phosphate; FEC—fluoroethylene carbonate; LLTZO—tantalum-doped lithium lanthanum zirconium oxide Li_6.5_La_3_Zr_1.5_Ta_0.5_O_12_; EGPEA—ethylene glycol phenyl ether acrylate; UiO-66—metal organic framework (MOF) made up of Zr_6_O_4_(OH)_4_ clusters with 1,4-benzodicarboxylic acid; LFP—lithium iron phosphate LiFePO_4_, LFMP—LiFe_0.15_Mn_0.85_PO_4_ ; NMC—nickel manganese cobalt oxide.

## References

[1] Danzi F., Salgado R.M., Oliveira J.E., Arteiro A., Camanho P.P., Braga M.H. (2021). Structural Batteries: A Review. Molecules.

[2] Sequeira C., Santos D. (2010). Introduction to Polymer Electrolyte Materials.

[3] Asp L., Johansson M., Lindbergh G., Xu J., Zenkert D. (2019). Structural Battery Composites: A Review. Funct. Compos. Struct..

[4] Hellqvist Kjell M. (2013). Performance of Conventional and Structural Lithium-Ion Batteries. Ph.D. Thesis.

[5] Zhou D., Shanmukaraj D., Tkacheva A., Armand M., Wang G. (2019). Polymer Electrolytes for Lithium-Based Batteries: Advances and Prospects. Chem.

[6] De Souza F.L., Leite E.R., Sequeira C., Santos D.B.T.P.E. (2010). Hybrid Polymer Electrolytes for Electrochemical Devices.

[7] Long L., Wang S., Xiao M., Meng Y. (2016). Polymer electrolytes for lithium polymer batteries. J. Mater. Chem. A.

[8] Mauger A., Armand M., Julien C.M., Zaghib K. (2017). Challenges and issues facing lithium metal for solid-state rechargeable batteries. J. Power Sources.

[9] Kotobuki M., Yan B., Pan F., Lu L., Savilov S., Aldoshin S. (2019). Low temperature sintering of crystallized Li_1.5_Al_0.5_Ge_1.5_(PO_4_)_3_ using hot-press technique. Mater. Today Proc..

[10] Kotobuki M., Suzuki Y., Kanamura K., Sato Y., Yamamoto K., Yoshida T. (2011). A novel structure of ceramics electrolyte for future lithium battery. J. Power Sources.

[11] Kotobuki M. (2020). Polymer Electrolytes. Polymer Electrolytes.

[12] Famprikis T., Canepa P., Dawson J.A., Islam M.S., Masquelier C. (2019). Fundamentals of inorganic solid-state electrolytes for batteries. Nat. Mater..

[13] Gebert F., Knott J., Gorkin R., Chou S.-L., Dou S.-X. (2021). Polymer electrolytes for sodium-ion batteries. Energy Storage Mater..

[14] Wu Y., Li Y., Wang Y., Liu Q., Chen Q., Chen M. (2022). Advances and prospects of PVDF based polymer electrolytes. J. Energy Chem..

[15] Or T., Gourley S.W.D., Kaliyappan K., Yu A., Chen Z. (2020). Recycling of mixed cathode lithium-ion batteries for electric vehicles: Current status and future outlook. Carbon Energy.

[16] Wright P.V. (1975). Electrical conductivity in ionic complexes of poly(ethylene oxide). Br. Polym. J..

[17] Fenton D.E., Parker J.M., Wright P.V. (1973). Complexes of alkali metal ions with poly(ethylene oxide). Polymer.

[18] Mindemark J., Lacey M.J., Bowden T., Brandell D. (2018). Beyond PEO—Alternative host materials for Li+-conducting solid polymer electrolytes. Prog. Polym. Sci..

[19] Armand M. (1983). Polymer solid electrolytes—An overview. Solid State Ion..

[20] Feuillade G., Perche P. (1975). Ion-conductive macromolecular gels and membranes for solid lithium cells. J. Appl. Electrochem..

[21] Skaarup S., West K., Zachau-Christiansen B. (1988). Mixed phase solid electrolytes. Solid State Ion..

[22] Wieczorek W., Such K., Wyciślik H., Płocharski J. (1989). Modifications of crystalline structure of peo polymer electrolytes with ceramic additives. Solid State Ion..

[23] Niu H., Wang L., Guan P., Zhang N., Yan C., Ding M., Guo X., Huang T., Hu X. (2021). Recent Advances in Application of Ionic Liquids in Electrolyte of Lithium Ion Batteries. J. Energy Storage.

[24] Li Q., Chen J., Fan L., Kong X., Lu Y. (2016). Progress in electrolytes for rechargeable Li-based batteries and beyond. Green Energy Environ..

[25] Chen Y., Kang Y., Zhao Y., Wang L., Liu J., Li Y., Liang Z., He X., Li X., Tavajohi N. (2021). A review of lithium-ion battery safety concerns: The issues, strategies, and testing standards. J. Energy Chem..

[26] Chen X., Li H., Yan Z., Cheng F., Chen J. (2019). Structure design and mechanism analysis of silicon anode for lithium-ion batteries. Sci. China Mater..

[27] Cai Y., Ku L., Wang L., Ma Y., Hongfei Z., Xu W., Han J., Qu B., Chen Y., Xie Q. (2019). Engineering oxygen vacancies in hierarchically Li-rich layered oxide porous microspheres for high-rate lithium ion battery cathode. Sci. China Mater..

[28] Mendes T.C., Zhang X., Wu Y., Howlett P.C., Forsyth M., Macfarlane D.R. (2019). Supported Ionic Liquid Gel Membrane Electrolytes for a Safe and Flexible Sodium Metal Battery. ACS Sustain. Chem. Eng..

[29] Xue Z., He D., Xie X. (2015). Poly(ethylene oxide)-based electrolytes for lithium-ion batteries. J. Mater. Chem. A.

[30] Xu K. (2004). Nonaqueous Liquid Electrolytes for Lithium-Based Rechargeable Batteries. Chem. Rev..

[31] Goodenough J.B., Kim Y. (2010). Challenges for Rechargeable Li Batteries. Chem. Mater..

[32] Aurbach D., Zinigrad E., Cohen Y., Teller H. (2002). A short review of failure mechanisms of lithium metal and lithiated graphite anodes in liquid electrolyte solutions. Solid State Ion..

[33] Zhang Q., Liu K., Ding F., Liu X. (2017). Recent advances in solid polymer electrolytes for lithium batteries. Nano Res..

[34] Selis L.A., Seminario J.M. (2018). Dendrite formation in silicon anodes of lithium-ion batteries. RSC Adv..

[35] Chou C.-Y., Hwang G.S. (2013). Surface effects on the structure and lithium behavior in lithiated silicon: A first principles study. Surf. Sci..

[36] Bieker G., Winter M., Bieker P. (2015). Electrochemical in situ investigations of SEI and dendrite formation on the lithium metal anode. Phys. Chem. Chem. Phys..

[37] Borzutzki K., Dong K., Nair J.R., Wolff B., Hausen F., Eichel R.-A., Winter M., Manke I., Brunklaus G. (2021). Lithium deposition in single-ion conducting polymer electrolytes. Cell Rep. Phys. Sci..

[38] Zhao Q., Stalin S., Zhao C.-Z., Archer L.A. (2020). Designing solid-state electrolytes for safe, energy-dense batteries. Nat. Rev. Mater..

[39] Tikekar M.D., Choudhury S., Tu Z., Archer L.A. (2016). Design principles for electrolytes and interfaces for stable lithium-metal batteries. Nat. Energy.

[40] Aziz S.B., Woo T.J., Kadir M.F.Z., Ahmed H.M. (2018). A conceptual review on polymer electrolytes and ion transport models. J. Sci. Adv. Mater. Devices.

[41] Berthier C., Gorecki W., Minier M., Armand M.B., Chabagno J.M., Rigaud P. (1983). Microscopic investigation of ionic conductivity in alkali metal salts-poly(ethylene oxide) adducts. Solid State Ion..

[42] Tarascon J.M., Armand M. (2001). Issues and challenges facing rechargeable lithium batteries. Nature.

[43] Ma Q., Zhang H., Zhou C., Zheng L., Cheng P., Nie J., Feng W., Hu Y.-S., Li H., Huang X. (2016). Single Lithium-Ion Conducting Polymer Electrolytes Based on a Super-Delocalized Polyanion. Angew. Chem. Int. Ed..

[44] Lin Y., Wang X., Liu J., Miller J.D. (2017). Natural halloysite nano-clay electrolyte for advanced all-solid-state lithium-sulfur batteries. Nano Energy.

[45] Zhao Q., Liu X., Stalin S., Khan K., Archer L.A. (2019). Solid-state polymer electrolytes with in-built fast interfacial transport for secondary lithium batteries. Nat. Energy.

[46] Zhu J., Zhang Z., Zhao S., Westover A.S., Belharouak I., Cao P.-F. (2021). Single-Ion Conducting Polymer Electrolytes for Solid-State Lithium–Metal Batteries: Design, Performance, and Challenges. Adv. Energy Mater..

[47] Nykaza J.R., Savage A., Pan Q., Wang S., Beyer F., Tang M.H., Li C.Y., Elabd Y.J.P. (2016). Polymerized ionic liquid diblock copolymer as solid-state electrolyte and separator in lithium-ion battery. Polymer.

[48] Pelz A., Dörr T.S., Zhang P., de Oliveira P.W., Winter M., Wiemhöfer H.-D., Kraus T. (2019). Self-Assembled Block Copolymer Electrolytes: Enabling Superior Ambient Cationic Conductivity and Electrochemical Stability. Chem. Mater..

[49] Yue L., Ma J., Zhang J., Zhao J., Dong S., Liu Z., Cui G., Chen L. (2016). All solid-state polymer electrolytes for high-performance lithium ion batteries. Energy Storage Mater..

[50] Ramesh S., Ng H.M. (2011). An investigation on PAN–PVC–LiTFSI based polymer electrolytes system. Solid State Ion..

[51] Wang L., Li X., Yang W. (2010). Enhancement of electrochemical properties of hot-pressed poly(ethylene oxide)-based nanocomposite polymer electrolyte films for all-solid-state lithium polymer batteries. Electrochim. Acta.

[52] Rivas B.L., Pereira E.D., Moreno-Villoslada I. (2003). Water-soluble polymer–metal ion interactions. Prog. Polym. Sci..

[53] Klongkan S., Pumchusak J. (2015). Effects of Nano Alumina and Plasticizers on Morphology, Ionic Conductivity, Thermal and Mechanical Properties of PEO-LiCF3SO3 Solid Polymer Electrolyte. Electrochim. Acta.

[54] Chaurasia S.K., Shalu, Gupta A.K., Verma Y.L., Singh V.K., Tripathi A.K., Saroj A.L., Singh R.K. (2015). Role of ionic liquid [BMIMPF6] in modifying the crystallization kinetics behavior of the polymer electrolyte PEO-LiClO4. RSC Adv..

[55] Wang Y., Zhong W.-H. (2015). Development of Electrolytes towards Achieving Safe and High-Performance Energy-Storage Devices: A Review. ChemElectroChem.

[56] Wang Y., Li B., Ji J., Eyler A., Zhong W.-H. (2013). A Gum-Like Electrolyte: Safety of a Solid, Performance of a Liquid. Adv. Energy Mater..

[57] Shen Y. (2004). Porous PVDF with LiClO_4_ complex as “solid” and “wet” polymer electrolyte. Solid State Ion..

[58] Jeong H.-S., Kim J.H., Lee S.-Y. (2010). A novel poly(vinylidene fluoride-hexafluoropropylene)/poly(ethylene terephthalate) composite nonwoven separator with phase inversion-controlled microporous structure for a lithium-ion battery. J. Mater. Chem..

[59] Subramania A., Kalyana Sundaram N.T., Vijaya Kumar G., Vasudevan T. (2006). New polymer electrolyte based on (PVA–PAN) blend for Li-ion battery applications. Ionics.

[60] Lin C., Hung C., Venkateswarlu M., Hwang B. (2005). Influence of TiO2 nano-particles on the transport properties of composite polymer electrolyte for lithium-ion batteries. J. Power Sources.

[61] Zhang Y., Lu W., Cong L., Liu J., Sun L., Mauger A., Julien C.M., Xie H., Liu J. (2019). Cross-linking network based on Poly(ethylene oxide): Solid polymer electrolyte for room temperature lithium battery. J. Power Sources.

[62] Sun C., Wang Z., Yin L., Xu S., Ghazi Z.A., Shi Y., An B., Sun Z., Cheng H.-M., Li F. (2020). Fast lithium ion transport in solid polymer electrolytes from polysulfide-bridged copolymers. Nano Energy.

[63] Kalnaus S., Asp L.E., Li J., Veith G.M., Nanda J., Daniel C., Chen X.C., Westover A., Dudney N.J. (2021). Multifunctional approaches for safe structural batteries. J. Energy Storage.

[64] Dias F.B., Plomp L., Veldhuis J.B.J. (2000). Trends in polymer electrolytes for secondary lithium batteries. J. Power Sources.

[65] Ding P., Lin Z., Guo X., Wu L., Wang Y., Guo H., Li L., Yu H. (2021). Polymer electrolytes and interfaces in solid-state lithium metal batteries. Mater. Today.

[66] Zhou X., Li X., Li Z., Xie H., Fu J., Wei L., Yang H., Guo X. (2021). Hybrid electrolytes with an ultrahigh Li-ion transference number for lithium-metal batteries with fast and stable charge/discharge capability. J. Mater. Chem. A.

[67] Silva M., Bermudez V., Pawlicka A. (2019). Insight on Polymer Electrolytes for Electrochemical Devices Applications.

[68] Hu J., Wang W.H., Zhu X.J., Liu S.B., Wang Y.J., Xu Y.J., Zhou S.K., He X.C., Xue Z.G. (2021). Composite polymer electrolytes reinforced by hollow silica nanotubes for lithium metal batteries. J. Membr. Sci..

[69] Cao J., He R., Kyu T. (2017). Fire retardant, superionic solid state polymer electrolyte membranes for lithium ion batteries. Curr. Opin. Chem. Eng..

[70] Johan M.R., Fen L.B. (2010). Combined effect of CuO nanofillers and DBP plasticizer on ionic conductivity enhancement in the solid polymer electrolyte PEO–LiCF3SO3. Ionics.

[71] Ye F., Liao K., Ran R., Shao Z. (2020). Recent Advances in Filler Engineering of Polymer Electrolytes for Solid-State Li-Ion Batteries: A Review. Energy Fuels.

[72] Huang H., Ding F., Zhong H., Li H., Zhang W., Liu X., Xu Q. (2018). Nano-SiO2-embedded poly(propylene carbonate)-based composite gel polymer electrolyte for lithium–sulfur batteries. J. Mater. Chem. A.

[73] Zhu Y., Cao J., Chen H., Yu Q., Li B. (2019). High electrochemical stability of a 3D cross-linked network PEO@nano-SiO_2_ composite polymer electrolyte for lithium metal batteries. J. Mater. Chem. A.

[74] Khurana R., Schaefer J.L., Archer L.A., Coates G.W. (2014). Suppression of lithium dendrite growth using cross-linked polyethylene/poly(ethylene oxide) electrolytes: A new approach for practical lithium-metal polymer batteries. J. Am. Chem. Soc..

[75] Wang H., Sheng L., Yasin G., Wang L., Xu H., He X. (2020). Reviewing the current status and development of polymer electrolytes for solid-state lithium batteries. Energy Storage Mater..

[76] Pan X., Yang P., Guo Y., Zhao K., Xi B., Lin F., Xiong S. (2021). Electrochemical and Nanomechanical Properties of TiO_2_ Ceramic Filler Li-Ion Composite Gel Polymer Electrolytes for Li Metal Batteries. Adv. Mater. Interfaces.

[77] Irfan M., Atif M., Yang Z., Zhang W. (2021). Recent advances in high performance conducting solid polymer electrolytes for lithium-ion batteries. J. Power Sources.

[78] Deng K., Zeng Q., Wang D., Liu Z., Qiu Z., Zhang Y., Xiao M., Meng Y. (2020). Single-ion conducting gel polymer electrolytes: Design, preparation and application. J. Mater. Chem. A.

[79] Putri R.M., Sundari C.D.D., Floweri O., Mayangsari T.R., Ivansyah A.L., Santosa S.P., Arcana I.M., Iskandar F. (2021). PEO/PVA/LiOH Solid Polymer Electrolyte Prepared via Ultrasound-assisted Solution Cast Method. J. Non-Cryst. Solids.

[80] Luo K., Shao D., Yang L., Liu L., Chen X., Zou C., Wang D., Luo Z., Wang X. (2021). Semi-interpenetrating gel polymer electrolyte based on PVDF-HFP for lithium ion batteries. J. Appl. Polym. Sci..

[81] Swiderska-Mocek A., Kubis A. (2021). Preparation and electrochemical properties of polymer electrolyte containing lithium difluoro(oxalato)borate or lithium bis(oxalate)borate for Li-ion polymer batteries. Solid State Ion..

[82] Gong X., Luo H., Liu G., Luo C., Niu Y., Li G. (2021). High-performance gel polymer electrolytes derived from PAN-POSS/PVDF composite membranes with ionic liquid for lithium ion batteries. Ionics.

[83] He Y., Liu N., Kohl P.A. (2021). Difunctional block copolymer with ion solvating and crosslinking sites as solid polymer electrolyte for lithium batteries. J. Power Sources.

[84] Cheng Z., Liu T., Zhao B., Shen F., Jin H., Han X. (2021). Recent advances in organic-inorganic composite solid electrolytes for all-solid-state lithium batteries. Energy Storage Mater..

[85] Zhang B., Liu Y., Pan X., Liu J., Doyle-Davis K., Sun L., Liu J., Jiao X., Jie J., Xie H. (2020). Dendrite-free lithium metal solid battery with a novel polyester based triblock copolymer solid-state electrolyte. Nano Energy.

[86] Mallela Y.L.N.K., Kim S., Seo G., Kim J.W., Kumar S., Lee J., Lee J.-S. (2020). Crosslinked poly(allyl glycidyl ether) with pendant nitrile groups as solid polymer electrolytes for Li–S batteries. Electrochim. Acta.

[87] Tseng Y.-C., Hsiang S.-H., Tsao C.-H., Teng H., Hou S.-S., Jan J.-S. (2021). In situ formation of polymer electrolytes using a dicationic imidazolium cross-linker for high-performance lithium ion batteries. J. Mater. Chem. A.

[88] Deng K., Zhou S., Xu Z., Xiao M., Meng Y. (2022). A high ion-conducting, self-healing and nonflammable polymer electrolyte with dynamic imine bonds for dendrite-free lithium metal batteries. Chem. Eng. J..

[89] Liang L., Yuan W., Chen X., Liao H. (2021). Flexible, nonflammable, highly conductive and high-safety double cross-linked poly(ionic liquid) as quasi-solid electrolyte for high performance lithium-ion batteries. Chem. Eng. J..

[90] Zeng Z., Chen X., Sun M., Jiang Z., Hu W., Yu C., Cheng S., Xie J. (2021). Nanophase-Separated, Elastic Epoxy Composite Thin Film as an Electrolyte for Stable Lithium Metal Batteries. Nano Lett..

[91] Liu Z., Chai J., Xu G., Wang Q., Cui G. (2015). Functional lithium borate salts and their potential application in high performance lithium batteries. Coord. Chem. Rev..

[92] Le Bideau J., Viau L., Vioux A. (2011). Ionogels, ionic liquid based hybrid materials. Chem. Soc. Rev..

[93] Hu P., Chai J., Duan Y., Liu Z., Cui G., Chen L. (2016). Progress in nitrile-based polymer electrolytes for high performance lithium batteries. J. Mater. Chem. A.

[94] Liu B., Huang Y., Cao H., Zhao L., Huang Y., Song A., Lin Y., Li X., Wang M. (2018). A novel porous gel polymer electrolyte based on poly(acrylonitrile-polyhedral oligomeric silsesquioxane) with high performances for lithium-ion batteries. J. Membr. Sci..

[95] Kim D.-G., Sohn H.-S., Kim S.-K., Lee A., Lee J.-C. (2012). Star-shaped polymers having side chain poss groups for solid polymer electrolytes; synthesis, thermal behavior, dimensional stability, and ionic conductivity. J. Polym. Sci. Part A Polym. Chem..

[96] Zhang J., Ma C., Liu J., Chen L., Pan A., Wei W. (2016). Solid polymer electrolyte membranes based on organic/inorganic nanocomposites with star-shaped structure for high performance lithium ion battery. J. Membr. Sci..

[97] Giles J., Gray F., MacCallum J., Vincent C. (1987). Synthesis and characterization of ABA block copolymer-based polymer electrolytes. Polymer.

[98] Gray F., MacCallum J., Vincent C., Giles J. (1988). Novel polymer electrolytes based on ABA block copolymers. Macromolecules.

[99] Zhou D., He Y.B., Liu R., Liu M., Du H., Li B., Cai Q., Yang Q.H., Kang F. (2015). In Situ Synthesis of a Hierarchical All-Solid-State Electrolyte Based on Nitrile Materials for High-Performance Lithium-Ion Batteries. Adv. Energy Mater..

[100] Cho Y.G., Hwang C., Cheong D.S., Kim Y.S., Song H.K. (2019). Gel/solid polymer electrolytes characterized by in situ gelation or polymerization for electrochemical energy systems. Adv. Mater..

[101] Han J.G., Kim K., Lee Y., Choi N.S. (2019). Scavenging Materials to Stabilize LiPF6-Containing Carbonate-Based Electrolytes for Li-Ion Batteries. Adv. Mater..

[102] Chen S., Che H., Feng F., Liao J., Wang H., Yin Y., Ma Z.-F. (2019). Poly (vinylene carbonate)-based composite polymer electrolyte with enhanced interfacial stability to realize high-performance room-temperature solid-state sodium batteries. ACS Appl. Mater. Interfaces.

[103] Karuppasamy K., Theerthagiri J., Vikraman D., Yim C.-J., Hussain S., Sharma R., Maiyalagan T., Qin J., Kim H.-S. (2020). Ionic Liquid-Based Electrolytes for Energy Storage Devices: A Brief Review on Their Limits and Applications. Polymers.

[104] Tian X., Yi Y., Yang P., Liu P., Qu L., Li M., Hu Y.-s., Yang B. (2019). High-Charge Density Polymerized Ionic Networks Boosting High Ionic Conductivity as Quasi-Solid Electrolytes for High-Voltage Batteries. ACS Appl. Mater. Interfaces.

[105] Lu Q., He Y.-B., Yu Q., Li B., Kaneti Y.V., Yao Y., Kang F., Yang Q.-H. (2017). Dendrite-Free, High-Rate, Long-Life Lithium Metal Batteries with a 3D Cross-Linked Network Polymer Electrolyte. Adv. Mater..

[106] Matsumoto K., Endo T. (2013). Design and synthesis of ionic-conductive epoxy-based networked polymers. React. Funct. Polym..

[107] Zhou J., Ji H., Liu J., Qian T., Yan C. (2019). A new high ionic conductive gel polymer electrolyte enables highly stable quasi-solid-state lithium sulfur battery. Energy Storage Mater..

[108] Andrews W.T., Liebig A., Cook J., Marsh P., Ciocanel C., Lindberg G.E., Browder C.C. (2018). Development of a PEO-based lithium ion conductive epoxy resin polymer electrolyte. Solid State Ion..

[109] Schulze M.W., McIntosh L.D., Hillmyer M.A., Lodge T.P. (2014). High-Modulus, High-Conductivity Nanostructured Polymer Electrolyte Membranes via Polymerization-Induced Phase Separation. Nano Lett..

[110] Xu J., Johannisson W., Johansen M., Liu F., Zenkert D., Lindbergh G., Asp L.E. (2020). Characterization of the adhesive properties between structural battery electrolytes and carbon fibers. Compos. Sci. Technol..

[111] Lim J.Y., Kang D.A., Kim N.U., Lee J.M., Kim J.H. (2019). Bicontinuously crosslinked polymer electrolyte membranes with high ion conductivity and mechanical strength. J. Membr. Sci..

[112] Bouchet R., Maria S., Meziane R., Aboulaich A., Lienafa L., Bonnet J.-P., Phan T.N.T., Bertin D., Gigmes D., Devaux D. (2013). Single-ion BAB triblock copolymers as highly efficient electrolytes for lithium-metal batteries. Nat. Mater..

[113] Jeong K., Park S., Lee S.-Y. (2019). Revisiting polymeric single lithium-ion conductors as an organic route for all-solid-state lithium ion and metal batteries. J. Mater. Chem. A.

[114] Tsuchida E., Ohno H., Kobayashi N., Ishizaka H. (1989). Poly[(ι-carboxy)oligo(oxyethylene) methacrylate] as a new type of polymeric solid electrolyte for alkali-metal ion transport. Macromolecules.

[115] Tada Y., Sato M., Takeno N., Nakacho Y., Shigehara K. (1994). Attempts at lithium single-ionic conduction by anchoring sulfonate anions as terminating groups of oligo(oxyethylene) side chains in comb-type polyphosphazenes. Chem. Mater..

[116] Gao J., Sun C., Xu L., Chen J., Wang C., Guo D., Chen H. (2018). Lithiated Nafion as polymer electrolyte for solid-state lithium sulfur batteries using carbon-sulfur composite cathode. J. Power Sources.

[117] Lee J., Song J., Lee H., Noh H., Kim Y.-J., Kwon S.H., Lee S.G., Kim H.-T. (2017). A Nanophase-Separated, Quasi-Solid-State Polymeric Single-Ion Conductor: Polysulfide Exclusion for Lithium–Sulfur Batteries. ACS Energy Lett..

[118] Siska D.P., Shriver D.F. (2001). Li+ Conductivity of Polysiloxane−Trifluoromethylsulfonamide Polyelectrolytes. Chem. Mater..

[119] Meziane R., Bonnet J.-P., Courty M., Djellab K., Armand M. (2011). Single-ion polymer electrolytes based on a delocalized polyanion for lithium batteries. Electrochim. Acta.

[120] Rohan R., Pareek K., Chen Z., Cai W., Zhang Y., Xu G., Gao Z., Cheng H. (2015). A high performance polysiloxane-based single ion conducting polymeric electrolyte membrane for application in lithium ion batteries. J. Mater. Chem. A.

[121] Zhu Y.S., Wang X.J., Hou Y.Y., Gao X.W., Liu L.L., Wu Y.P., Shimizu M. (2013). A new single-ion polymer electrolyte based on polyvinyl alcohol for lithium ion batteries. Electrochim. Acta.

[122] Xu G., Zhang Y., Rohan R., Cai W., Cheng H. (2014). Synthesis, Characterization and Battery Performance of A Lithium Poly (4-vinylphenol) Phenolate Borate Composite Membrane. Electrochim. Acta.

[123] He Y., Liu N., Kohl P.A. (2021). Lithium Ion Conduction in Diblock Polymer Electrolyte with Tethered Anion. ChemistrySelect.

[124] Wang C., Li R., Chen P., Fu Y., Ma X., Shen T., Zhou B., Chen K., Fu J., Bao X. (2021). Highly stretchable, non-flammable and notch-insensitive intrinsic self-healing solid-state polymer electrolyte for stable and safe flexible lithium batteries. J. Mater. Chem. A.

[125] Li D., Luo L., Zhu J., Qin H., Liu P., Sun Z., Lei Y., Jiang M. (2021). A hybrid lithium sulfonated polyoxadiazole derived single-ion conducting gel polymer electrolyte enabled effective suppression of dendritic lithium growth. Chin. Chem. Lett..

[126] Cao C., Li Y., Feng Y., Peng C., Li Z., Feng W. (2019). A solid-state single-ion polymer electrolyte with ultrahigh ionic conductivity for dendrite-free lithium metal batteries. Energy Storage Mater..

[127] Pan Q., Jiang S., Li Z., Liu Y., Du Y., Zhao N., Zhang Y., Liu J.-M. (2021). Highly porous single ion conducting membrane via a facile combined “structural self-assembly” and in-situ polymerization process for high performance lithium metal batteries. J. Membr. Sci..

[128] Zhang J., Wang S., Han D., Xiao M., Sun L., Meng Y. (2020). Lithium (4-styrenesulfonyl) (trifluoromethanesulfonyl) imide based single-ion polymer electrolyte with superior battery performance. Energy Storage Mater..

[129] Zhang Y., Wang J., Tan C., He Y., Chen Y., Huo S., Zeng D., Li C., Cheng H. (2021). Fire-retardant sp3 boron-based single ion conducting polymer electrolyte for safe, high efficiency and dendrite-free Li-metal batteries. J. Membr. Sci..

[130] Cao C., Li Y., Feng Y., Long P., An H., Qin C., Han J., Li S., Feng W. (2017). A sulfonimide-based alternating copolymer as a single-ion polymer electrolyte for high-performance lithium-ion batteries. J. Mater. Chem. A.

[131] Van Humbeck J.F., Aubrey M.L., Alsbaiee A., Ameloot R., Coates G.W., Dichtel W.R., Long J.R. (2015). Tetraarylborate polymer networks as single-ion conducting solid electrolytes. Chem. Sci..

[132] Shin D.-M., Bachman J.E., Taylor M.K., Kamcev J., Park J.G., Ziebel M.E., Velasquez E., Jarenwattananon N.N., Sethi G.K., Cui Y. (2020). A Single-Ion Conducting Borate Network Polymer as a Viable Quasi-Solid Electrolyte for Lithium Metal Batteries. Adv. Mater..

[133] Weston J.E., Steele B.C.H. (1982). Effects of inert fillers on the mechanical and electrochemical properties of lithium salt-poly(ethylene oxide) polymer electrolytes. Solid State Ion..

[134] Fan P., Liu H., Marosz V., Samuels N.T., Suib S.L., Sun L., Liao L. (2021). High Performance Composite Polymer Electrolytes for Lithium-Ion Batteries. Adv. Funct. Mater..

[135] Hoang Huy V.P., So S., Hur J. (2021). Inorganic Fillers in Composite Gel Polymer Electrolytes for High-Performance Lithium and Non-Lithium Polymer Batteries. Nanomaterials.

[136] Shen Z., Cheng Y., Sun S., Ke X., Liu L., Shi Z. (2021). The critical role of inorganic nanofillers in solid polymer composite electrolyte for Li^+^ transportation. Carbon Energy.

[137] Zhang D., Xu X., Qin Y., Ji S., Huo Y., Wang Z., Liu Z., Shen J., Liu J. (2020). Recent Progress in Organic–Inorganic Composite Solid Electrolytes for All-Solid-State Lithium Batteries. Chem.—A Eur. J..

[138] Zhao Y., Wang L., Zhou Y., Liang Z., Tavajohi N., Li B., Li T. (2021). Solid Polymer Electrolytes with High Conductivity and Transference Number of Li Ions for Li-Based Rechargeable Batteries. Adv. Sci..

[139] Sasikumar M., Krishna R.H., Raja M., Therese H.A., Balakrishnan N.T.M., Raghavan P., Sivakumar P. (2021). Titanium dioxide nano-ceramic filler in solid polymer electrolytes: Strategy towards suppressed dendrite formation and enhanced electrochemical performance for safe lithium ion batteries. J. Alloys Compd..

[140] Zhan H., Wu M., Wang R., Wu S., Li H., Tian T., Tang H. (2021). Excellent Performances of Composite Polymer Electrolytes with Porous Vinyl-Functionalized SiO2 Nanoparticles for Lithium Metal Batteries. Polymers.

[141] Xu H.M., Jing M.X., Li J., Huang Z.H., Wang T.F., Yuan W.Y., Ju B.W., Shen X.Q. (2021). Safety-Enhanced Flexible Polypropylene Oxide-ZrO2 Composite Solid Electrolyte Film with High Room-Temperature Ionic Conductivity. ACS Sustain. Chem. Eng..

[142] Liu M., Zhang S., Li G., Wang C., Li B., Li M., Wang Y., Ming H., Wen Y., Qiu J. (2021). A cross-linked gel polymer electrolyte employing cellulose acetate matrix and layered boron nitride filler prepared via in situ thermal polymerization. J. Power Sources.

[143] Sun Y., Wang J., Fu D., Zhang F., Wang Z., Chen X., Xu J., Hu J., Wu X. (2021). Flexible Composite Solid Electrolyte with an Active Inorganic Filler. ACS Sustain. Chem. Eng..

[144] Zhou P., Yao D., Zuo K., Xia Y., Yin J., Liang H., Zeng Y.-P. (2021). Highly dispersible silicon nitride whiskers in asymmetric porous separators for high-performance lithium-ion battery. J. Membr. Sci..

[145] Tian L., Li A., Huang Q., Zhang Y., Long D. (2021). Homogenously dispersed ultrasmall niobium(V) oxide nanoparticles enabling improved ionic conductivity and interfacial compatibility of composite polymer electrolyte. J. Colloid Interface Sci..

[146] Hu J., Chen K., Yao Z., Li C. (2021). Unlocking solid-state conversion batteries reinforced by hierarchical microsphere stacked polymer electrolyte. Sci. Bull..

[147] Li Z., Liu F., Chen S., Zhai F., Li Y., Feng Y., Feng W. (2021). Single Li ion conducting solid-state polymer electrolytes based on carbon quantum dots for Li-metal batteries. Nano Energy.

[148] Asp L.E., Bouton K., Carlstedt D., Duan S., Harnden R., Johannisson W., Johansen M., Johansson M.K.G., Lindbergh G., Liu F. (2021). A Structural Battery and its Multifunctional Performance. Adv. Energy Sustain. Res..

[149] Jamal H., Khan F., Hyun S., Min S.W., Kim J.H. (2021). Enhancement of the ionic conductivity of a composite polymer electrolyte via surface functionalization of SSZ-13 zeolite for all-solid-state Li-metal batteries. J. Mater. Chem. A.

[150] Hu S., Du L., Zhang G., Zou W., Zhu Z., Xu L., Mai L. (2021). Open-Structured Nanotubes with Three-Dimensional Ion-Accessible Pathways for Enhanced Li+ Conductivity in Composite Solid Electrolytes. ACS Appl. Mater. Interfaces.

[151] Jin Y., Zong X., Zhang X., Liu C., Li D., Jia Z., Li G., Zhou X., Wei J., Xiong Y. (2021). Interface regulation enabling three-dimensional Li_1.3_Al_0.3_Ti_1.7_(PO_4_)_3_-reinforced composite solid electrolyte for high-performance lithium batteries. J. Power Sources.

[152] Walle K.Z., Musuvadhi Babulal L., Wu S.H., Chien W.C., Jose R., Lue S.J., Chang J.K., Yang C.C. (2021). Electrochemical Characteristics of a Polymer/Garnet Trilayer Composite Electrolyte for Solid-State Lithium-Metal Batteries. ACS Appl. Mater Interfaces.

[153] Zhang Z., Huang Y., Zhang G., Chao L. (2021). Three–dimensional fiber network reinforced polymer electrolyte for dendrite–free all–solid–state lithium metal batteries. Energy Storage Mater..

[154] Shen X., Li R., Ma H., Peng L., Huang B., Zhang P., Zhao J. (2020). Enhancing Li+ transport kinetics of PEO-based polymer electrolyte with mesoporous silica-derived fillers for lithium-ion batteries. Solid State Ion..

[155] He H., Chai Y., Zhang X., Shi P., Fan J., Xu Q., Min Y. (2021). A 2D–3D co-conduction effect in PEO-based all-solid-state batteries for long term cycle stability. J. Mater. Chem. A.

[156] Barbosa J.C., Gonçalves R., Costa C.M., de Zea Bermudez V., Fidalgo-Marijuan A., Zhang Q., Lanceros-Méndez S. (2021). Metal–organic frameworks and zeolite materials as active fillers for lithium-ion battery solid polymer electrolytes. Mater. Adv..

[157] Zhang Z., Huang Y., Gao H., Li C., Hang J., Liu P. (2021). MOF-derived multifunctional filler reinforced polymer electrolyte for solid-state lithium batteries. J. Energy Chem..

[158] Wu X., Chen K., Yao Z., Hu J., Huang M., Meng J., Ma S., Wu T., Cui Y., Li C. (2021). Metal organic framework reinforced polymer electrolyte with high cation transference number to enable dendrite-free solid state Li metal conversion batteries. J. Power Sources.

[159] Zhang Z., Huang Y., Li C., Li X. (2021). Metal–Organic Framework-Supported Poly(ethylene oxide) Composite Gel Polymer Electrolytes for High-Performance Lithium/Sodium Metal Batteries. ACS Appl. Mater. Interfaces.

[160] Lv F., Liu K., Wang Z., Zhu J., Zhao Y., Yuan S. (2021). Ultraviolet-cured polyethylene oxide-based composite electrolyte enabling stable cycling of lithium battery at low temperature. J. Colloid Interface Sci..

[161] Kuai Y., Wang F., Yang J., Lu H., Xu Z., Xu X., NuLi Y., Wang J. (2021). Silica-nanoresin crosslinked composite polymer electrolyte for ambient-temperature all-solid-state lithium batteries. Mater. Chem. Front..

[162] Didwal P.N., Singhbabu Y.N., Verma R., Sung B.-J., Lee G.-H., Lee J.-S., Chang D.R., Park C.-J. (2021). An advanced solid polymer electrolyte composed of poly(propylene carbonate) and mesoporous silica nanoparticles for use in all-solid-state lithium-ion batteries. Energy Storage Mater..

[163] Wu J., Chen J., Wang X., Zhou A., Yang Z. (2021). Applying multi-scale silica-like three-dimensional networks in a PEO matrix via in situ crosslinking for high-performance solid composite electrolytes. Mater. Chem. Front..

[164] Zhang X., Guo W., Zhou L., Xu Q., Min Y. (2021). Surface-modified boron nitride as a filler to achieve high thermal stability of polymer solid-state lithium-metal batteries. J. Mater. Chem. A.

[165] Ramachandran M., Subadevi R., Rajkumar P., Muthupradeepa R., Sivakumar M. (2021). Electrochemical analyses of ZrO_2_ dispersoid incorporated poly (styrene-methyl methacrylate) blend gel electrolytes for lithium-ion battery. J. Appl. Polym. Sci..

[166] Beshahwured S.L., Wu Y.-S., Wu S.-h., Chien W.-C., Jose R., Lue S.J., Yang C.-C. (2021). Flexible hybrid solid electrolyte incorporating ligament-shaped Li_6.25_Al_0.25_La_3_Zr_2_O_12_ filler for all-solid-state lithium-metal batteries. Electrochim. Acta.

[167] Chen F., Jing M.-X., Yang H., Yuan W.-Y., Liu M.-Q., Ji Y.-S., Hussain S., Shen X.-Q. (2021). Improved ionic conductivity and Li dendrite suppression of PVDF-based solid electrolyte membrane by LLZO incorporation and mechanical reinforcement. Ionics.

[168] Mu S., Huang W., Sun W., Zhao N., Jia M., Bi Z., Guo X. (2021). Heterogeneous electrolyte membranes enabling double-side stable interfaces for solid lithium batteries. J. Energy Chem..

[169] Yu X., Liu Y., Goodenough J.B., Manthiram A. (2021). Rationally Designed PEGDA–LLZTO Composite Electrolyte for Solid-State Lithium Batteries. ACS Appl. Mater. Interfaces.

[170] Guan D., Huang Y., He M., Hu G., Peng Z., Cao Y., Du K. (2021). Multilayer PEO/LLZTO composite electrolyte enables high-performance solid-state Li-ion batteries. Ionics.

[171] Luo S., Zhao E., Gu Y., Huang J., Zhang Z., Yang L., Hirano S.-I. (2021). Rational design of fireproof fiber-network reinforced 3D composite solid electrolyte for dendrite-free solid-state batteries. Chem. Eng. J..

[172] Siyal S.H., Shah S.S.A., Najam T., Javed M.S., Imran M., Lan J.-L. (2021). Significant Reduction in Interface Resistance and Super-Enhanced Performance of Lithium-Metal Battery by In Situ Construction of Poly(vinylidene fluoride)-Based Solid-State Membrane with Dual Ceramic Fillers. ACS Appl. Energy Mater..

[173] Wu X., Zheng Y., Li W., Liu Y., Zhang Y., Li Y., Li C. (2021). Solid electrolytes reinforced by infinite coordination polymer nano-network for dendrite-free lithium metal batteries. Energy Storage Mater..

[174] Carlstedt D., Asp L.E. (2019). Thermal and diffusion induced stresses in a structural battery under galvanostatic cycling. Compos. Sci. Technol..

[175] Snyder J.F., Carter R.H., Wetzel E.D. (2007). Electrochemical and Mechanical Behavior in Mechanically Robust Solid Polymer Electrolytes for Use in Multifunctional Structural Batteries. Chem. Mater..

[176] Guigon M., Oberlin A., Desarmot G. (1984). Microtexture and structure of some high-modulus, PAN-base carbon fibres. Fibre Sci. Technol..

[177] Tanaka F., Okabe T., Okuda H., Kinloch I.A., Young R.J. (2014). Factors controlling the strength of carbon fibres in tension. Compos. Part A Appl. Sci. Manuf..

[178] Snyder J.F., Wong E.L., Hubbard C.W. (2009). Evaluation of Commercially Available Carbon Fibers, Fabrics, and Papers for Potential Use in Multifunctional Energy Storage Applications. J. Electrochem. Soc..

[179] Snyder J., O’Brien D., Baechle D., Mattson D., Wetzel E. Structural Composite Capacitors, Supercapacitors, and Batteries for U.S. Army Applications. Proceedings of the ASME 2008 Conference on Smart Materials, Adaptive Structures and Intelligent Systems. Smart Materials, Adaptive Structures and Intelligent Systems.

[180] Liu P., Sherman E., Jacobsen A. (2009). Design and fabrication of multifunctional structural batteries. J. Power Sources.

[181] Ekstedt S., Wysocki M., Asp L. (2010). Structural batteries made from fibre reinforced composites. Plast. Rubber Compos..

[182] Danzi F., Camanho P.P., Braga M.H. (2021). An all-solid-state coaxial structural battery using sodium-based electrolyte. Molecules.

[183] Salgado R.M., Danzi F., Oliveira J.E., El-Azab A., Camanho P.P., Braga M.H. (2021). The latest trends in Electric Vehicles batteries. Molecules.

[184] Lv F., Wang Z., Shi L., Zhu J., Edström K., Mindemark J., Yuan S. (2019). Challenges and development of composite solid-state electrolytes for high-performance lithium ion batteries. J. Power Sources.

[185] Duan H., Fan M., Chen W.-P., Li J.-Y., Wang P.-F., Wang W.-P., Shi J.-L., Yin Y.-X., Wan L.-J., Guo Y.-G. (2019). Extended Electrochemical Window of Solid Electrolytes via Heterogeneous Multilayered Structure for High-Voltage Lithium Metal Batteries. Adv. Mater..

[186] Yao P., Zhu B., Zhai H., Liao X., Zhu Y., Xu W., Cheng Q., Jayyosi C., Li Z., Zhu J. (2018). PVDF/Palygorskite Nanowire Composite Electrolyte for 4 V Rechargeable Lithium Batteries with High Energy Density. Nano Lett.

[187] Xu D., Su J., Jin J., Sun C., Ruan Y., Chen C., Wen Z. (2019). In Situ Generated Fireproof Gel Polymer Electrolyte with Li6.4Ga0.2La3Zr2O12 As Initiator and Ion-Conductive Filler. Adv. Energy Mater..

[188] Li Z., Sha W.-X., Guo X. (2019). Three-Dimensional Garnet Framework-Reinforced Solid Composite Electrolytes with High Lithium-Ion Conductivity and Excellent Stability. ACS Appl. Mater. Interfaces.

[189] Niu C., Liu J., Chen G., Liu C., Qian T., Zhang J., Cao B., Shang W., Chen Y., Han J. (2019). Anion-regulated solid polymer electrolyte enhances the stable deposition of lithium ion for lithium metal batteries. J. Power Sources.

[190] Huo H., Wu B., Zhang T., Zheng X., Ge L., Xu T., Guo X., Sun X. (2019). Anion-immobilized polymer electrolyte achieved by cationic metal-organic framework filler for dendrite-free solid-state batteries. Energy Storage Mater..

[191] Sun Z., Li Y., Zhang S., Shi L., Wu H., Bu H., Ding S. (2019). g-C3N4 nanosheets enhanced solid polymer electrolytes with excellent electrochemical performance, mechanical properties, and thermal stability. J. Mater. Chem. A.

[192] Ihrner N., Johannisson W., Sieland F., Zenkert D., Johansson M. (2017). Structural lithium ion battery electrolytes via reaction induced phase-separation. J. Mater. Chem. A.

[193] Schneider L.M., Ihrner N., Zenkert D., Johansson M. (2019). Bicontinuous Electrolytes via Thermally Initiated Polymerization for Structural Lithium Ion Batteries. ACS Appl. Energy Mater..

[194] Moyer K., Meng C., Marshall B., Assal O., Eaves J., Perez D., Karkkainen R., Roberson L., Pint C.L. (2020). Carbon fiber reinforced structural lithium-ion battery composite: Multifunctional power integration for CubeSats. Energy Storage Mater..

